# The Role of Exosomes Derived From Mesenchymal Stromal Cells in Dermatology

**DOI:** 10.3389/fcell.2021.647012

**Published:** 2021-04-07

**Authors:** María I. Quiñones-Vico, Raquel Sanabria-de la Torre, Manuel Sánchez-Díaz, Álvaro Sierra-Sánchez, Trinidad Montero-Vílchez, Ana Fernández-González, Salvador Arias-Santiago

**Affiliations:** ^1^Cell Production and Tissue Engineering Unit, Virgen de las Nieves University Hospital, Granada, Spain; ^2^Granada Biosanitary Research Institute (ibs. GRANADA), Granada, Spain; ^3^Andalusian Network for the Design and Translation of Advanced Therapies, Seville, Spain; ^4^Department of Dermatology, School of Medicine, University of Granada, Granada, Spain; ^5^Department of Dermatology, Virgen de las Nieves University Hospital, Granada, Spain

**Keywords:** exosomes-based therapy, immunomodulation, mesenchymal stem cell-derived exosomes, regenerative medicine, skin autoimmune diseases, skin wound healing

## Abstract

The skin is the largest organ of the human body and its main functions include providing protection from external harmful agents, regulating body temperature, and homeostatic maintenance. Skin injuries can damage this important barrier and its functions so research focuses on approaches to accelerate wound healing and treat inflammatory skin diseases. Due to their regenerative and immunomodulatory properties, mesenchymal stromal cells (MSCs) have been reported to play a significant role in skin repair and regeneration. However, it seems that the secretome of these cells and exosomes in particular may be responsible for their functions in skin regeneration and the immunomodulation field. The present review aims to gather the available information about the role of MSC-derived exosomes for both *in vitro* and *in vivo* models of different skin conditions and to highlight the need for further research in order to overcome any limitations for clinical translation.

## Introduction

The skin is the largest organ of the human body and constitutes a protective barrier that isolates us from harmful agents and injuries. Apart from its defensive function against physical, chemical, and biological agents, the skin also contributes to regulating the organism’s temperature, homeostatic maintenance, participation in the mechanisms of sensorial perception, as well as in regenerative processes (Oualla-Bachiri et al., 2020; Sanabria-de la Torre et al., 2020).

The skin is frequently damaged as a result of acute and chronic wounds such as extensive burns, trauma or diabetic ulcers and also because of other conditions like atopic dermatitis (AD), aging or oxidative stress (Cho et al., 2018; Lai et al., 2018; Roşca et al., 2018; Casado-Díaz et al., 2020; Zhang Y. et al., 2020). Patients with cutaneous wounds experience physical and mental health problems, and these wounds also have a huge socioeconomic burden (Wu et al., 2018). Recently, mesenchymal stromal cells (MSCs) have gained much attention in cutaneous repair and regeneration. Resident skin MSCs are actively involved in the wound-healing process, either by differentiating into fibroblasts, which are responsible for the matrix synthesis, or through the release of various molecules involved in tissue regeneration, such as anti-scarring, anti-apoptotic and pro-angiogenic factors. Thus, the paracrine activity of resident and recruited cells effects the suitability of the regenerative process (Ballini et al., 2018). In fact, several studies have applied exogenous MSCs to wounds in order to benefit from their regenerative properties, resulting in positive effects on both wound healing and scarring (Roşca et al., 2018). This therapeutic potential of MSCs is mainly due to their facility to be isolated and expanded *in vitro* and the possibility of being cryopreserved once isolated, without significant loss of their potential. Furthermore, MSCs are hypoimmunogenic since these cells express intermediate or low levels of the MHC class I and II molecules respectively (Casado-Díaz et al., 2020). MSCs can be isolated from different tissues, although the most widely used are bone marrow-derived MSCs (BM-MSCs), adipose tissue-derived MSCs (AT-MSCs), and umbilical cord-derived MSCs (UC-MSCs). Another promising source of MSCs is the oral cavity, including tissues such as dental pulp, human periapical inflamed cyst, dental pulp of human exfoliated deciduous teeth, periodontal ligament, dental follicle progenitors, root apical papilla of human teeth and gingiva (Spagnuolo et al., 2018). Moreover, MSCs have been isolated from other regions such as amniotic fluid, the periosteum and fetal tissues, all of which show phenotypic heterogeneity (Hoang et al., 2020). They can also be extracted from the blood, liver, spleen, and bone marrow of the human fetus in the first and second trimesters (Hoang et al., 2020).

However, despite the significant progress which has been made in the application of MSCs in wound repair and cutaneous regeneration, there are some limitations inherent to MSC cell therapy. For instance, there is considerable heterogeneity in the delivery protocols and MSC populations which makes it difficult to determine the impact of timing of delivery, number of cells produced and site of delivery on MSCs. In addition, there is no evidence that MSCs differentiate into phenotypes typical of resident cutaneous cells during skin wound healing (Hu et al., 2019). Lastly, current challenges for the use of MSCs concern the lack of universally accepted criteria for defining the MSC phenotype and their functional properties. Actually, MSCs mediate distinct immune modulating responses that are characterized by a pro-inflammatory MSC1 phenotype and an immunosuppressive MSC2 phenotype (Lai et al., 2018). Other limitations include their proliferation capacity, lifespan, potential contamination by handling and rejection (Ferreira and Gomes, 2019).

There is current evidence that MSCs achieve a therapeutic effect *in vivo* mainly through paracrine signaling (Wu et al., 2018; Hu et al., 2019; Casado-Díaz et al., 2020) and not only due to their capacity to proliferate and differentiate into the required cellular types in the damaged tissue but also because of their secretome (Ballini et al., 2017). The MSC– secretome has one free fraction, made of soluble factors and metabolites, and another encapsulated into extracellular vesicles (EVs).

Extracellular vesicles are typically classified into three subtypes according to size and biogenesis mechanisms: exosomes (50–100 nm), microvesicles (100–1000 nm), and apoptotic bodies (500–5000 nm) (Qiu et al., 2019). Exosomes are generated through endocytosis, from larger intracellular vesicles called multivesicular bodies (MVBs) through a sophisticated intracellular trafficking system (Aheget et al., 2020). MVBs are intraluminal vesicles, formed by internal budding of the endosomal membrane. The best-known mechanism of MVB and exosome generation is that carried out by the endosomal sorting complex required for transport (ESCRT), although other mechanisms such as hydrolysis of sphingomyelin into ceramides or proteins like tetraspanins have been reported (Andreu and Yáñez-Mó, 2014). MVBs migrate toward the edge of the cell where they fuse with the plasma membrane and exosomes are then released to the extracellular space via exocytosis (Qiu et al., 2019; Casado-Díaz et al., 2020). Thus, exosome biogenesis can be divided into three stages: the formation of endocytic vesicles, through the invagination of the plasma membrane; the formation of MVBs, by the inward budding of the endosomal membranes; and the fusion of MVBs with the plasma membrane and exosome release (Casado-Díaz et al., 2020). In addition to MSC surface antigens such as CD90, CD73, and CD105, exosomes have multiple specific marker proteins, including membrane transport and fusion proteins (GTPases, annexins, and flotillin), tetraspannins (CD9, CD63, and CD81), heat shock proteins (hsp60, hsp70, and hsp90), proteins involved in MVB biogenesis (Alix and tumor susceptibility gene 101 protein), as well as lipid-related proteins and phospholipases (Liu et al., 2018; Ferreira and Gomes, 2019; Casado-Díaz et al., 2020).

Exosomes are key bioactive vesicles responsible for the paracrine effects of MSCs, regulating many physiological and pathological processes by affecting the survival, proliferation, migration and gene expression of recipient cells and by reprogramming targeted cell behaviors (Wu et al., 2018). Several studies have indicated that some exosomes are involved in the skin’s physiological and pathological processes (Liu et al., 2018). Compared to MSC therapy, MSC exosomes have the following advantages: Firstly, MSC exosomes exert intense biological effects because of their direct fusion with target cells. Secondly, MSC exosomes can be stored and transported at −70°C for long periods of time since their effective components are protected by their membrane, which is not easily destroyed (Zhao et al., 2017). Thirdly, the concentration, dose, route and time of use are easy to control. Lastly, there is no risk of the immune rejection and tumorigenesis caused by cell transplantation therapy (Hu et al., 2019; An et al., 2020; Yu et al., 2020). In addition, exosomes can be sterilized by filtration during their preparation for clinical usage (Lai et al., 2018). Regarding exosome administration routes, the most common are intravenous and subcutaneous injections although they can also be administered through biocompatible scaffolds or hydrogels, which can serve as sustained release systems for these vesicles (Wang C. et al., 2019; Yang J. et al., 2020).

**FIGURE 1:**
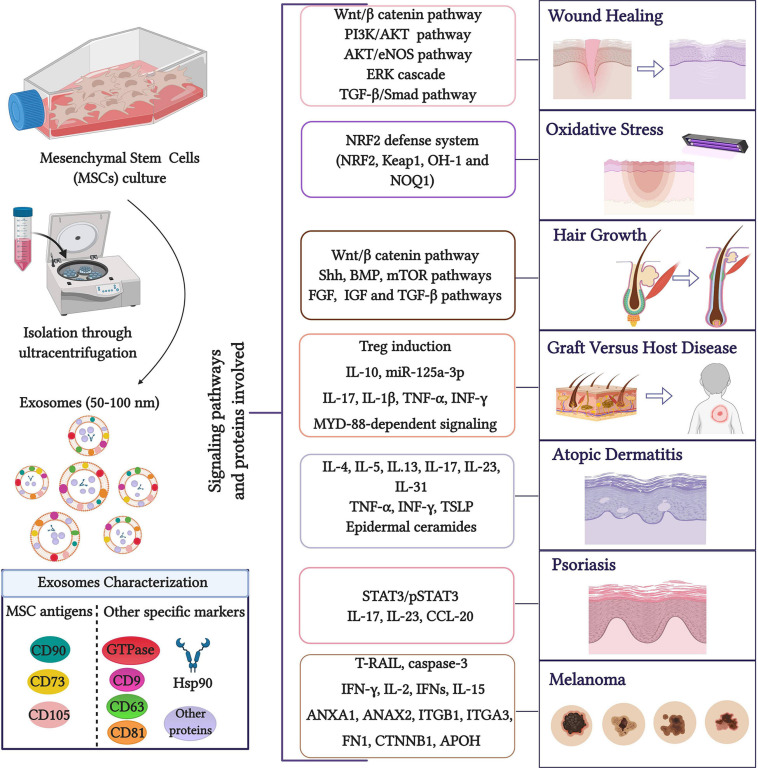
Role of MSC exosomes in dermatology. Exosomes are widely isolated through MSC culture media ultracentrifugation (Gardiner et al., 2016) although other isolation methods such as commercial kits, polyethylene glycol/polymer-based EV enrichment and size-based fractionation have been described (Witwer et al., 2019). The presence of MSC exosome markers can be analyzed by methods including Western blotting, enzyme-linked immunosorbent assays, classical flow cytometry of bead-captured EVs, or advanced flow cytometry at the single EV level. The main signaling pathways and proteins involved in the role of MSC exosomes are highlighted. Created with BioRender.com.

Due to the current interest in the potential therapeutic applications of MSC-derived exosomes, the main objective of the present review is to analyze their role in dermatology, focusing on wound healing and skin regeneration, **GVHD**, AD, psoriasis, photoaging, oxidative stress, and rejuvenation (Figure 1).

## Exosomes and Wound Healing

Cutaneous wound healing is a complex, dynamic process in charge of restoring the structure and function of damaged tissues. It involves highly orchestrated multiple processes including hemostasis, inflammation, cell migration and proliferation, angiogenesis and extracellular matrix remodeling (Lai et al., 2018; Gao et al., 2020) which are strictly regulated by multiple diverse growth factors, cytokines, enzymes and structural matrix proteins generated by multiple cell types such as dermal fibroblasts, epidermal keratinocytes, and immune cells (Zhao et al., 2017; Hoang et al., 2020). Among the varied factors influencing wound repair, angiogenesis occupies a critical position, which results in the delivery of nutrients and oxygen to the wound sites, promoting fibroblast proliferation, collagen synthesis, and re-epithelialization (Liu et al., 2020; Yu et al., 2020). Under pathological conditions, the disruption and prolonging of the wound-healing process can lead to chronic, non-healing wounds such as diabetic wounds (Liu et al., 2020). The mechanisms underlying poor healing of diabetic wounds are still unclear although the main complications involve hypoxia, impaired angiogenesis, damage from reactive oxygen species (ROS) and neuropathy, leading to long-term medical burden and compromised quality of life in those patients (Wang C. et al., 2019; An et al., 2020).

Another problem associated with impaired wound healing is scarring. Cutaneous scar formation is a consequence of the wound-healing process and involves the coordination of a complex sequence of interactions between cells, ECM components and signaling molecules. Scar tissue is characterized by the exaggerated deposition of ECM components and a lack of skin appendages such as hair follicles and sweat glands. ECM remodeling, especially collagen synthesis and degradation, is the key cellular and molecular event contributing to scarring. Furthermore, the fibroblast-myofibroblast transition is critical in this process. In response to skin injury and wound damage, dermal fibroblasts undergo a phenotype transition into myofibroblasts, characterized by enhanced contractile ability and the expression of α-smooth muscle actin (α-SMA). Moreover, several studies have stated that the Transforming Growth Factor (TGF)-β1/Smad signaling pathway actively takes part in collagen formation and the fibroblast-myofibroblast transition (Wang et al., 2017). Interestingly, scarless healing occurs in the early or midgestation stages of embryonic development. Thus in fetal wound tissue, the ratio of collagen type III to type I is higher and there is also a higher ratio of TGF-β3 to TGF-β1 and of matrix metalloproteinases (MMPs) to tissue inhibitors of metalloproteinases (TIMPs).

Therapies based on MSCs showed great potential for wound healing due to their ability to recruit cells and release growth factors and proteins. In fact, MSCs have been tested as a promising cell-based therapy for diabetic wounds *in vitro* and *in vivo* because of their ability to accelerate wound closure with increased epithelialization, granulation tissue formation, and angiogenesis by differentiation into skin cells and paracrine pathways to repair injured cells. However, due to the disadvantages of MSCs, more efforts have been focused on the MSC secretome including cytokines, growth factors, chemokines and extracellular vesicles containing mRNA, proteins and microRNAs, as well as their role on the wound-healing process (An et al., 2020).

Mesenchymal stromal cell-derived exosomes can promote angiogenesis, cell migration, proliferation and the re-epithelialization process by activating a signaling route such as the Wnt/β-catenin, phosphatidylinositol 3-kinase/protein kinase B pathway (PI3K/AKT) or extracellular signal-regulated kinase (ERK) cascade, resulting in growth factors expression upregulation (Carrasco et al., 2019; Kucharzewski et al., 2019).

For instance, exosomes isolated from human AT-MSCs (hAT-MSCs) stimulate cell proliferation and migration and play an inhibitory role in the cell apoptosis of *in vitro* cultured human keratinocytes (HaCaTs) treated with hydrogen peroxide (H_2_O_2_) through Wnt/β-catenin signaling (Ma et al., 2019). Metastasis Associated Lung Adenocarcinoma Transcript 1 (MALAT1), a transcriptional regulator for numerous genes contained in these exosomes, can also mediate H_2_O_2_-induced wound healing by targeting miR-124 and activating this pathway (He et al., 2020). Zhang et al. showed that 14-3-3ζ protein in human UC-MSC (hUC-MSC) derived exosomes enhances the Hippo/Yes-associated protein (YAP) pathway, promoting self-regulation of Wnt/β-catenin signaling at the remodeling phase of cutaneous regeneration *in vivo* and restricting excessive skin cell expansion and collagen deposition 4 weeks after treatment in a rat deep second-degree burn model (Zhang et al., 2016). They found that exosomal 14-3-3ζ promoted the binding of p-LATS (large tumor suppressor) and YAP at high cell density, resulting in phosphorylation of YAP. Interestingly, miR-135a-mediated downregulation of large tumor suppressor 2 (LATS2) increased the migration of BJ cells *in vitro* and promoted wound healing *in vivo* (Gao et al., 2020). Regarding Wnt/β-catenin signaling, Wnt4 contained in hUC-MSC-derived exosomes promoted β-catenin nuclear translocation and activity to enhance proliferation and migration of HaCaTs *in vitro* and played a key role in wound re-epithelialization in a rat skin burn model *in vivo* (Zhang et al., 2015a). β-catenin nuclear translocation induced the increased expression of proliferating cell nuclear antigen (PCNA), cyclin D3, *N*-cadherin and β-catenin and the decreased expression of *E*-cadherin. Furthermore, Wnt4 induces β-catenin activation in endothelial cells and exerts proangiogenic effects *in vitro*, which could be an important mechanism for cutaneous wound healing (Zhang et al., 2015b). Other proliferative markers, growth factors and migration-related chemokines such as angiotensin-2 (Ang-2) (Liu et al., 2020), cyclin D1, cyclin A2 and C-X-C motif chemokine 12 (CXCL12) were significantly upregulated after hUC-MSCs-derived exosome treatments with human umbilical vein endothelial cells (HUVECs) *in vitro*, promoting proliferation, migration and angiogenesis (Li X. et al., 2020). In this study, *in vivo* administration of iron oxide nanoparticle-labeled exosomes significantly increased the number of exosomes accumulated at the injury site, enhancing endothelial cell proliferation, migration and angiogenic tubule formation as well as reducing scar formation due to the increased expression of CK19, PCNA, and collagen.

Another important signaling route in the wound-healing process, parallel to the Wnt/β-catenin pathway, is PI3K/AKT. Yang C. et al. (2020) reported that miR-21 packaged in hAT-MSC-derived exosomes enhances MMP-9 expression and decreases TIMP-1 through the PI3K/AKT pathway, promoting the proliferation of HaCaTs *in vitro*. Moreover, miR-126-mediated phosphatase and tensin homolog (PTEN) downregulation seems to stimulate angiogenesis *in vitro* through the PI3K/AKT pathway, contributing to the stimulation of wound healing and angiogenesis in diabetic rats *in vivo* (Ding et al., 2019). This route is also reported to be involved in promoting fibroblast proliferation and the optimization of collagen deposition *in vitro* after hAT-MSC-derived exosome treatment. *In vivo*, these exosomes significantly accelerated wound healing (Zhang W. et al., 2018). Regarding AKT, Yu et al. (2020) showed that exosomes from human BM-MSCs (hBM-MSCs) treated with atorvastatin (ATV) activated AKT/endothelial nitric oxide synthase (eNOS) signaling pathway to augment the angiogenesis of endothelial cells via upregulating miR-211-3p, resulting in accelerated wound regeneration in diabetic rats *in vivo*. The same effect has been reported after treatment with exosomes from the same origin pre-treated with serum of neonatal and adult mice (Qiu et al., 2020). In addition to AKT, hBM-MSC-derived exosomes are able to activate ERK1/2 and Signal transducer and activator of transcription 3 (STAT3), promoting the expression of trophic factors such as cyclin D2 *in vitro*. This stimulates fibroblast growth, migration and angiogenesis of endothelial cells *in vitro* (Shabbir et al., 2015). Induced mesenchymal stromal cell (iMSC) derived exosomes have also been proven to stimulate ERK1/2 thus promoting the proliferation of HaCaTs and human dermal fibroblasts (HDFs) *in vivo* (Kim et al., 2018). Moreover, exosomes obtained from hBM-MSCs, hAT-MSCs, and hUC-MSCs have been reported to be able to induce HaCaT and HDF proliferation and migration *in vitro* by inducing crucial wound healing-mediated growth factors (Choi et al., 2018) such as vascular endothelial growth factor A (VEGF-A), fibroblast growth factor 2 (FGF-2), hepatocyte growth factor (HGF) and platelet-derived growth factor BB (PDGF-BB), which can activate AKT, ERK, and STAT3 signaling (Hoang et al., 2020). Continuing with ERK proteins, Wang et al. (2017) reported that hAT-MSC-derived exosomes increased the MMP3 expression of skin dermal fibroblasts by activating the ERK/Mitogen-activated protein kinase (MAPK) pathway, leading to a high ratio of MMP3 to TIMP1, which is also beneficial for ECM remodeling. These exosomes also decreased the size of scars and increased the ratio of collagen III to collagen I in murine incisional wounds *in vivo* by preventing the differentiation of fibroblasts into myofibroblasts and increasing the ratio of TGF-β3 to TGF-β1 (Wang et al., 2017).

Regarding scarless wound healing, several studies have noted the role of the TGF-β/Smad signaling pathway. For instance, Jiang T. et al. (2020) showed that hBM-MSC-derived exosomes promoted HaCaT and HDF growth *in vitro* and accelerated scarless wound healing *in vivo*. They found significantly downregulated TGF-β1, Smad2, Smad3, and Smad4 expression, alongside upregulated TGF-β3 and Smad7 expression (Jiang T. et al., 2020). Furthermore, exosomes from hUC-MSCs reduced scar formation and myofibroblast accumulation in a skin defect mouse model *in vivo*. Specifically, Fang et al. (2016) reported that these exosomes contain specific microRNAs such as miR-21, miR-23a, miR-125b, and miR-145 which play key roles in suppressing myofibroblast formation by inhibiting the transforming TGF-β2/Smad2 pathway. In line with this, Jiang L. et al. (2020). demonstrated that tumor necrosis factor-inducible gene 6 (TSG-6) overexpressed hBM-MSC-derived exosomes effectively ameliorated scar pathological injury, decreased inflammatory molecular secretion and attenuated collagen deposition in a mouse skin wound model *in vivo*. The exosome treatment downregulated the expression of TGF-β1, p-Smad2 and p-Smad3 in the scar region (Jiang L. et al., 2020). Smad2/3 phosphorylation has also been found markedly decreased in fibroblasts treated with hUC-MSC-derived exosomes along with Collagen I and III and α-SMA (Hu et al., 2020). Considering the collagen I/III ratio, Dalirfardouei et al. (2019) reported that exosomes originating from human menstrual MSCs (hMen-MSCs) rapidly decreased this ratio leading to less scar formation in a diabetic mouse model and promoting re-epithelialization, wound closure and angiogenesis *in vivo*. Exosomes from hAT-MSCs exerted the same effects by increasing gene expression of *N*-cadherin, cyclin-1, PCNA and collagen I, III *in vitro* and collagen I and III production in the early stage of wound healing *in vivo*. Interestingly, in the late stage, exosomes inhibited collagen expression to reduce scar formation (Hu et al., 2016). In addition, human amniotic epithelial cell (hAEC) derived exosomes remarkably enhanced the proliferation and migration ability of fibroblasts *in vitro* and collagen I and collagen III were downregulated in these cells through stimulating the expression of MMP-1. *In vivo* wound assays also showed that exosome treatment facilitated the wound-healing process with well-arranged collagen fibers (Zhao et al., 2017).

Other signaling routes reported to be involved in the wound-healing process are the poly ADP ribose polymerase 1(PARP-1)/apoptosis-inducing factor (AIF) apoptosis pathway and Notch signaling pathway. The first seems to suppress HaCaT apoptosis induced with H_2_O_2_ by inhibiting nuclear translocation of AIF and upregulating PARP-1 after treatment with hUC-MSC-derived exosomes *in vitro*. *In vivo* experiments showed enhanced epidermal re-epithelialization and dermal angiogenesis (Zhao et al., 2020). Regarding the Notch signaling pathway, an *in vivo* study indicated that human fetal dermal mesenchymal stromal cell (hFD-MSC) derived exosomes could accelerate wound closure in a mouse full-thickness skin wound model by activating this route (Wang X. et al., 2019).

It should be noted that the administration route of exosomes in the *in vivo* studies described previously are mainly intravenous and subcutaneous injections of exosome solutions. However, some investigations reported hydrogel development as an efficient administration pathway. For example, a chitosan/silk hydrogel loaded with human gingival mesenchymal stromal cell (hG-MSC) derived exosomes accelerated skin defect healing *in vivo* (Shi et al., 2017) and a thermosensitive Pluronic F-127 (PF-127) hydrogel containing hUC-MSC-derived exosomes significantly accelerated wound closure rate in a diabetic animal model *in vivo* (Yang J. et al., 2020). Along with oxidative hyaluronic acid and poly-e-L-lysin (denoted as FHE hydrogel), PF-127 also significantly improved neovascularization and re-epithelialization in diabetic rats (Wang C. et al., 2019). Supplementary Tables 1, 2 summarize *in vitro* and *in vivo* studies respectively.

## Exosomes and Oxidative Stress, Photoaging and Rejuvenation

Some studies have analyzed whether MSC-derived exosomes can enhance skin rejuvenation by preventing oxidative stress and photoaging effects. Oxidative stress is a major cause of skin injury induced by ultraviolet (UV) irradiation and other stimuli which can damage the cellular lipids, proteins and DNA of skin cells by promoting the production of ROS and decreasing antioxidant enzyme activity. This results in sunburn, premature aging, and carcinogenesis. Keratinocytes constitute a barrier against environmental damage by modulating oxidative stress, glucose metabolism and inflammatory mediators through the nuclear factor E2-related factor 2 (NRF2) signaling pathway, which is a central player in regulating the expression of antioxidant enzymes following skin injury or inflammation (Schäfer et al., 2012; Wang et al., 2020). Wang et al. (2020) investigated the effects of hUC-MSC-derived exosomes on oxidative injury in H_2_O_2_-stimulated epidermal keratinocytes *in vitro* and a UV-irradiated mouse model *in vivo*. They found reduced ROS generation, DNA damage, aberrant calcium signaling and mitochondrial changes in addition to alleviated cellular and histological responses to inflammation and oxidation (Table 1). Furthermore, the NRF2 signaling pathway was involved in this antioxidation activity since its knockdown attenuated the antioxidant capacities of exosomes both *in vitro* and *in vivo* (Wang et al., 2020) (Table 1 and Supplementary Table 3).

**TABLE 1:** *In vitro* studies of MSC exosomes in other skin conditions.

**Source of exosomes**	**Isolation protocol**	***In vitro* model**	**Disease**	**Signaling pathway and proteins involved**	**Outcomes**	**References**
hUC-MSCs	The culture medium was processed using a series of centrifugation steps (300 *g* for 10 min, 2,000 *g* for 10 min and 10,000 *g* for 30 min). Exosomes were collected via ultracentrifugation at 100,000 g for 70 min	H_2_O_2_- stimulated primary keratinocyte culture	Oxidative stress	↓NRF2 Keap1, HO-1 and NQO1 (NRF2 defense system)	↓ROS generation and DNA damage ↓Aberrant calcium signaling ↓Mitochondrial changes	Wang et al., 2020
hUC-MSCs	The culture medium was successively centrifuged at 400 g for 10 min, 2,000 g for 30 min and 10,000 g for 60 min. The supernatant was passed through a syringe filter (0.22 μm) and centrifuged at 100,000 *g* for 120 min to pellet exosomes. The pellet was washed and then centrifuged for another 120 min at the same high speed	UV-irradiated HDFs	Rejuvenation	↑Collagen I, elastin and fibronectin ↓MMP-1	Suppressive effects against UV-induced damage	Zhang Y. et al., 2020a
hUC-MSCs	The culture medium was centrifuged at 300 *g* for 10 min and 16,500 *g* for 30 min and then filtered through a 0.22 μm filter. The final supernatant was then ultracentrifuged at 100,000 *g* for 70 min to pellet exosomes. The pellet was filtered through a 0.22 μm filter and centrifuged at the same speed	Dendritic cells HaCaTs	Psoriasis	↓IL-23 secretion ↓STAT3/pSTAT3 ↓IL-17, IL-23 and CCL20	Suppressed dendritic cell maturation and activation ↓Inflammatory responses	Zhang Y. et al., 2020b
hBM-MSCs	The culture medium was centrifuged at 2,000 g for 30 min. The supernatant was passed through a 0.2 μm filter, mixed with Total Exosome Isolation Reagent (Invitrogen/Thermo Fisher Scientific), incubated overnight and centrifuged at 10,000 *g* for 1 h	Peripheral blood mononuclear cells	aGVHD	↓Blocked T cell activation ↑miR-125a-3p	Immunoregulatory effects	Fujii et al., 2018
hBM-MSCs	The culture medium was centrifuged 200 g for 10 min, 2,000 g for 20 min, 10,000 g for 30 min and 110,000 g for 7 h at 4°C, followed by filtration using a 0.22 μm filter. The culture supernatant was collected and ultracentrifugation was performed with the same sequential centrifugation procedure. The pellet was washed twice and then filtered through the 0.22 μm filter	Peripheral blood mononuclear cells	cGVHD	Blocked Th17 differentiation ↑Improved Treg phenotype	Regulatory effects on cGVHD effector cells	Lai et al., 2018
hESC-MSCs	The culture medium was 0.22-μm filtered and concentrated 100 × for exosomes by tangential flow filtration (MWCO 100 kDa)	CD4^+^ T cells	GVHD	↑Polarization of CD4^+^ T cells to CD4^+^ CD25^+^ FoxP3^+^ in the presence of allogenic CD11c^+^ cells	Immunoregulatory effects	Zhang W. et al., 2018a
BM-MSCs	The culture medium was filtered through a 0.22 μm pore size membrane and subjected to three continuous centrifugations at 300 g (10 min), 1,200 g (30 min) and 10,000 g (45 min). Then the supernatant was concentrated using 100 kDa Amicon^®^ filter and exosomes were isolated with Exoquick TC kit according to the protocol	Melanoma murine cells	Melanoma	Not characterized	TRAIL exosomes induced higher apoptosis rate compared to non-modified exosomes	Shamili et al., 2018
irUC-MSCs	Sequential centrifugations. Not specified	Melanoma cell line A375	Melanoma	Not characterized	↓Cell survival rate	de Araujo Farias et al., 2018
BM-MSCs	The culture medium was centrifuged at 300 *g* for 10 min, at 1,500 *g* for 20 min and finally at 2,500 *g* for 20 min. The supernatant was filtered through a 0.2 μm syringe filter and ultracentrifuged at 100,000 g for 60 min. Pellets were washed and ultracentrifuged again	hDP-cells	Hair growth	↑AKT phosphorylation ↑Bcl2 ↑VEGF and IGF-1	↑Proliferation and migration ↑Hair growth	Rajendran et al., 2017
hUC-MSCs	Exosomes were isolated using exoEasy Maxi kit. Prefiltered culture medium was mixed with a binding buffer and added to the exoEasy membrane affinity column to bind the exosomes to the membrane. After centrifugation, the flow-through was discarded and wash buffer was added to the column. After another centrifugation and discarding the flow-through, the vesicles were eluted by adding elution buffer to the spin column, and the eluate was collected by centrifugation	HDFs Abdominal skin tissue (*ex vivo*)	Rejuvenation	↓MMP1	↑Proliferation and collagen synthesis (*in vitro*) ↑Collagen I and elastin synthesis (*ex vivo*)	Kim et al., 2017

In terms of skin rejuvenation, the ECM and especially collagen and elastin play a crucial role in skin growth and elasticity. Collagen is responsible for the mechanical protection of the body, prevention of skin dehydration, elasticity maintenance, tissue firmness and skin wrinkle minimization. Elastin is a major structural protein of body tissue and a fibrous protein which provides strength and natural elasticity (Kim et al., 2017). Kim and colleagues studied the capacity of hUC-MSC-derived exosomes to rejuvenate skin by modulating collagen production and permeation (Kim et al., 2017; Ferreira and Gomes, 2019). They reported stimulation of HDF migration and collagen synthesis *in vitro* and promotion of collagen I and elastin production in *ex vivo* human skin (Kim et al., 2017) (Table 1 and Supplementary Table 3). Interestingly, they also found decreased MMP-1 production, which works as a collagenase and encodes a secreted enzyme which breaks down the types I, II, and III interstitial collagens (Tallant et al., 2010; Kim et al., 2017). Lastly, Zhang K. et al. (2020) studied whether the marine sponge *Haliclona* sp. spicules (SHSs) could effectively enhance the skin delivery of exosomes from hUC-MSCs by evaluating its topical application in rejuvenating photoaged mouse skin. It was reported that the combination of exosomes and SHSs showed significant anti-photoaging effects in mice, including reducing microwrinkles, alleviating histopathological changes and promoting the expression of extracellular matrix constituents. Moreover, skin irritation tests on a guinea pig animal model showed slight irritation (Zhang K. et al., 2020) (Table 1 and Supplementary Table 3).

Taken together, these investigations prove the significant role of exosomes, mainly derived from hUC-MSCs, in preventing oxidative skin damage and promoting rejuvenation. This could lead to the potential use of MSC-derived exosomes in the development and application of cosmetics, although further studies about their effects on *in vivo* human skin are required.

## Exosomes and Hair Growth

Alopecia is a common medical problem which has serious negative impacts on affected individuals. It is related to various factors including nutritional deficiencies, hormonal changes and drug treatment (Rajendran et al., 2017). Alopecia areata and androgenic alopecia are challenging conditions for dermatologists nowadays, with a lack of effective treatments. Advanced therapies are a promising therapeutic option and have shown good results (Martinez-Lopez et al., 2020).

Various dermatological compounds have been developed to combat alopecia. Finasteride and minoxidil are the mainstay treatments for alopecia. However, they only have a short-term effect and discontinuation of treatment leads to rapid hair loss and has known side effects (Mysore, 2012; Suchonwanit et al., 2019; Ha et al., 2020). Autologous follicle transplantation has also been tried, but the number of donor follicles is limited, it is an invasive procedure and the graft survival rate largely depends on the surgeon. Therefore, alopecia is an unsolved problem requiring effective long-term strategies (Rajendran et al., 2017).

The hair follicle cycle is a complex process involving alternating phases of rapid growth (anagen), regression (catagen), and quiescence (telogen) (Alonso and Fuchs, 2006). Hair follicles are epidermal appendages that contain both epithelial and mesenchymal compartments. The dermal papilla (DP) is located at the base of the hair follicle and plays a fundamental role in the hair follicle cycle (Kishimoto et al., 2000; Rajendran et al., 2017). AT-MSCs are able to promote the proliferation of DP cells *in vitro* and promote hair growth in mice and humans (Fukuoka et al., 2017; Ha et al., 2020).

The Wnt/β-catenin signaling and Shh signaling pathways, in addition to the secretion of growth factors such as FGF-5 or insulin-like growth factor-1 (IGF-1), are crucial for hair follicle development and hair growth (Ha et al., 2020). Other routes involved in the telogen to anagen transition include the estrogen receptor pathway, Bone Morphogenetic Protein (BMP) signaling, mammalian Target of Rapamycin (mTOR) signaling, FGF and TGF-β signaling pathways (Rajendran et al., 2017).

Direct transplantation of DP cells has been tested and their ability to induce follicles has been demonstrated (Weinberg et al., 1993). However, cell transplantation therapy is generally associated with the risk of tumor formation, graft rejection and ethical concerns. DP-MSC-derived exosomes have been reported to induce the passage from telogen to anagen, as well as delayed transition from anagen to catagen *in vivo*. Furthermore, these exosomes stimulated the expression of β-catenin and Shh, regulators of the hair follicle cycle and the proliferation and migration of outer root sheath keratinocytes, increasing their entry into S and S/G1 phase. The result was the formation of hair shafts of greater length and diameter (Zhou et al., 2018). Moreover, Kwack et al. (2019) stated that DP-exosomes were able to induce the development of *in vitro* cultured human hair follicles and also hair growth *in vivo* mice models. Interestingly, Yan et al. (2019) found 111 miRNAs that are differentially expressed between DP cells and DP exosomes. There were 34 major miRNAs that pointed to exosomes as regulators of hair follicle stem cell proliferation and differentiation. Specifically, miR22-5p inhibits hair follicle stem cell proliferation by targeting the Lymphoid enhancer-binding factor 1 (LEF1) gene, an important regulator of the β-catenin signaling pathway (Yan et al., 2019).

Rajendran et al. (2017) have produced the only publication to date on MSC exosomes, where the effect of BM-MSC-derived exosomes on hair growth was studied (Rajendran et al., 2017) (Table 1 and Supplementary Table 3). They found that *in vitro* treatment of DP cells with these exosomes caused activation by phosphorylation of AKT, as well as of the antiapoptotic protein Bcl-2, thus increasing their survival and migration. On the other hand, the expression and release of VEGF and IGF-1 genes were significantly increased in a dose-dependent manner by treatment with MSC-EV. These factors promote hair growth, an increase in follicle size and hair thickness. *In vivo* effects of these exosomes led to an increase in Wnt3a and Wnt5a signaling, so the treatment could be useful for activating human hair follicle stem cells, resulting in anagen initiation through Wnt/β-catenin activation (Rajendran et al., 2017). BM-MSC exosomes significantly promoted the conversion of telogen to anagen and increased the thickness of the dermis, which also indirectly reflects the improvement of hair growth. Additionally, no major organ damage was observed, indicating that BM-MSC exosomes could be a non-toxic treatment option (Rajendran et al., 2017).

Alopecia areata and androgenic alopecia are challenging conditions for dermatologists nowadays, with a lack of effective treatments. Advanced therapies are a promising therapeutic option that have shown good results (Martinez-Lopez et al., 2020). Although few studies have focused on the use of exosomes to stimulate hair growth, the findings so far are promising. In fact, a patented study points to MSC exosomes as the central component of a pharmaceutical composition aimed at promoting hair growth (Lim et al., 2015). It is very likely that the role of MSC exosomes in hair follicle dynamics will become a high-impact tool in skin regenerating cosmetics and biomedicine (Carrasco et al., 2019).

## Exosomes and Graft Versus Host Disease

Graft versus host disease (GVHD) occurs when donor cells attack host cells. There are two ways of developing GVHD: acute GVHD (aGVHD) which appears earlier and usually remains as a skin rash, and chronic GVHD (cGVHD) which appears later and may affect more tissues and organs. cGVHD is the primary cause of long-term morbidity and mortality after allogeneic hematopoietic stem cell transplantation (Lai et al., 2018). The epithelial target tissues affected by classic cGVHD are the gastrointestinal tract, liver, skin, and lungs. In fact, pulmonary complications are currently considered diagnostic evidence of cGVHD and are characterized by a frequent lack of response to treatment and irreversibility. However, other systems such as the oral, esophageal, musculoskeletal, joint, fascial, ocular and lymphohematopoietic can also be damaged. A proposed conceptual model divides the pathophysiology of cGVHD into three stages: early inflammation (phase 1), later chronic inflammation, thymic injury and dysregulated B cell and T cell immunity (phase 2), and finally tissue repair with fibrosis (phase 3) (Zeiser and Blazar, 2017).

Firstly, the translocation of bacteria and fungi to the tissue creates damage that causes the release of pathogen-associated molecular patterns (PAMPs), as well as damage-associated molecular patterns (DAMPs), leading to a cascade of activation of toll-like receptors (TLRs), nucleotide-binding oligomerization domain-like receptors (NOD-R) and the NOD-like receptor protein 3 inflammasome (NLRP3) (Zeiser and Blazar, 2017). As the intima of the vessels is damaged by inflammation, endothelial cells are lost and the microvasculature is disorganized. In addition, T cells are activated during the initiation phase of chronic GVHD.

In the second stage, T cells are polarized toward type 1, type 2, and type 17 helper T (Th1, Th2, and Th17) cells. In addition, thymic epithelial cells are lost, which are necessary for the generation of regulatory T (Treg) cells and for positive selection of T lymphocytes (Zeiser and Blazar, 2017). The Th17/Treg ratio has been considered a specific marker of cGVHD progression (Lai et al., 2018).

Lastly, platelet-derived growth factor α (PDGF-α) and TGF-β activate fibroblasts, causing extracellular matrix production and ultimately the sclerotic phenotype. In addition, the production of isotype-switched immunoglobulin by differentiated B cells results in pathogenic immunoglobulin deposition in various organs, which contributes to organ damage and fibrosis (Zeiser and Blazar, 2017). The chronic inflammatory state is maintained by the Th17 cells that escaped immune regulation in the second stage (MacDonald et al., 2017).

Due to their immunomodulatory properties, MSCs are a promising therapy for preventing cGVHD. MSCs regulate the Th17/Treg balance and promote transportation tolerance (Weng et al., 2012). MSC exosomes mediate the paracrine effects of MSCs and promote tissue repair and homeostasis recovery, making them potential candidates for cell-free therapies (Lai et al., 2018). MSC exosomes are able to inhibit inflammation by suppressing the activation and migration of autoreactive T cells. Exosomes secreted by immune and non-immune cells can stimulate and inhibit the immune system. The effects of exosomes on immunity include the activation of T cells, polarization of T cells into Treg cells, immunosuppression, anti-inflammatory and others (Wang W. M. et al., 2019).

*In vivo* and *in vitro* experiments have demonstrated the ability of MSC exosomes to inhibit cGVHD by promoting the expansion of Treg cells whilst inhibiting pro-inflammatory Th17 cells (Table 1 and Supplementary Table 3). In fact, hBM-MSCs-derived exosomes exert marked immunosuppressive effects on cytokine production (Lai et al., 2018). Specifically, these exosomes inhibit the expression of pro-inflammatory cytokines such as IL-17A, a characteristic cytokine of Th17 cells. Furthermore, they are capable of inducing the production of IL-10, a characteristic cytokine of Treg. In this way, hBM-MSCs-derived exosomes carry out regulatory immune responses capable of attenuating cGVHD. Also, they are a promising therapeutic tool for treating pulmonary complications in cGVHD (Lai et al., 2018).

Several studies reveal the effective immunomodulatory potential of MSC exosomes for the treatment of cGVHD by regulating Treg through different pathways, such as MYD88-dependent signaling (Zhang et al., 2014) (Supplementary Table 3) or by pathways dependent on antigen-presenting cells. Furthermore, it appears that induction of Tregs mediated by human embryonic stem cell–derived MSCs (hESC-MSCs)-derived-exosomes requires previous T cell activation (Zhang B. et al., 2018) (Table 1 and Supplementary Table 3). The immunosuppressive effect of MSC exosomes has been evaluated in a mouse model of myocardial ischemia/reperfusion injury, renal fibrosis, liver injury, etc. (Lai et al., 2010; Lee et al., 2012; Li et al., 2013). Furthermore, hBM-MSC-derived exosomes had immunoregulatory effects on peripheral blood mononuclear cells *in vitro* and could prolong the survival of mice with aGVHD and improve aGvHD damage *in vivo* (Fujii et al., 2018) (Table 1 and Supplementary Table 3).

In a preliminary clinical study, a patient with resistant grade IV aGVHD was treated with hBM-MSC-derived exosomes (Kordelas et al., 2014) (Supplementary Table 3). The levels of IL-1β, TNF-α, and IFN-γ produced by peripheral blood mononucleated cells (PBMC) were reduced by more than 50% after the last application. In this context, the clinical outcome of aGVHD improved significantly shortly after the start of MSC exosome therapy (Kordelas et al., 2014). Therefore, MSC-derived exosomes may potentially provide a new and safe tool for treating therapy-refractory GVHD and other diseases potentially associated with inflammation (Leung, 2013).

In addition to GVHD, allograft rejection is the main reason for the failure of organ transplantation. A novel study analyzed the role of exosomes as RNA transport vehicles to induce immune tolerance in patients with skin grafts. The RNA delivery system of targeted dendritic cells (DC exosomes) was constructed based on MSC exosomes. DC exosomes were able to induce immune tolerance 3, 7, and 14 days after skin transplantation. Furthermore, the long-term immune tolerance of the graft was maintained in the murine model (Li C. et al., 2020).

## Exosomes and Atopic Dermatitis

Chronic uncontrolled inflammatory responses are associated with various inflammatory diseases, including allergic skin diseases such as AD (Shin et al., 2020). Due to their intrinsic immunosuppressive properties, MSCs are a key element in the regulation of inflammation and therefore in the treatment of these allergic diseases. Furthermore, the clinical value of MSCs in AD has been confirmed in clinical trials (phase I/IIa) (Carrasco et al., 2019). Specifically, MSC exosomes represent an excellent alternative to MSC cell therapy since MSC exosomes have biological functions similar to those of parent cells, while they are more stable and have lower immunogenicity. The anti-inflammatory and immunomodulatory functions of MSC exosomes have been widely described (Ha et al., 2020).

Specifically, AD is a chronic skin disease with serious erythematous lesions and severe systemic inflammation. AD is related to genetic, immunological and environmental factors and its prevalence is higher in developed countries, particularly in recent years (Sanabria-de la Torre et al., 2020). Different factors must be taken into account in order to develop an effective treatment against AD. On one hand, elevated levels of Th2 cytokines are associated with abnormal immune responses that increase susceptibility to AD (Leung, 2000). On the other hand, the abnormal expression of genes responsible for epidermal barrier function is a crucial factor in the development of AD (Silverberg and Silverberg, 2015). The pathogenesis of AD includes changes in the skin barrier, abnormal immune signaling and defective terminal differentiation of keratinocytes, leading to decreased levels of ceramides, filaggrin and antimicrobial peptides (Leung, 2013). Alteration of the skin barrier leads to severe skin inflammation, allowing the entry of pathogens, allergens and toxic environmental pollutants. Ceramides contribute to keratinocyte differentiation and thus epidermal barrier function (Mizutani et al., 2009). In fact, AD patients have reduced ceramide levels (Imokawa et al., 1990).

Regarding the epidermal barrier in AD, Shin et al. (2020) demonstrate that hAT-MSC-derived exosomes are capable of improving the barrier functions of epidermal permeability, which coincides with a significant increase in ceramides and a reduction in immune responses during AD progression (Supplementary Table 3). In addition, MSC exosomes stimulated the production of epidermal ceramides and the formation of laminar bilayers at the stratum granulosa-stratum corneum interface, which contributes to the differentiation of keratinocytes and helps form an adequate epidermal permeability barrier. Finally, MSC exosomes were also found to activate genes associated with keratinocyte differentiation (Shin et al., 2020). Therefore, MSC exosomes offer a promising cell-free therapeutic option for the treatment of AD, thanks to their role in the *de novo* synthesis of ceramides, in the activation of genes involved in keratinocyte differentiation, lipid metabolism, cell cycle as well as their intervention in the regulation of the immune response.

Regarding their role as mediators of inflammation, hAT-MSC-derived exosomes significantly decreased the production of pro-inflammatory cytokines [Interleukin (IL) −4, 5, 13, 17], tumor necrosis factor α (TNF-α), interferon gamma (IFN-γ) and thymic stromal lymphopoeitin (TSLP) in the murine model of AD in a dose-dependent manner. Since TSLP is also known to be an important itch inducer, this result implied that AT-MSC exosomes helped reduce itching (Shin et al., 2020) (Supplementary Table 3). Moreover, MSC exosomes promote anti-inflammatory polarization of M2 macrophages and reduce pro-inflammatory polarization of M1 macrophages. In this way, MSC exosomes reduce the expression of Th2 cytokines, including IL-4, IL-13, IL-23, and IL-31, therapeutic targets for AD (Cho et al., 2018; Ha et al., 2020; Shin et al., 2020). In fact, Cho et al. (2018) studied the intravenous (IV) and subcutaneous (SC) administration of hAT-MSC-derived exosomes finding AD symptoms significantly decreased in a dose-dependent manner in both murine models (Supplementary Table 3). These exosomes were able to reduce the clinical score, the numbers of MCs, CD86+ cells and CD206+ cells in the dermal tissue, as well as the mRNA levels of associated inflammatory factors (Cho et al., 2018).

## Exosomes and Psoriasis

Psoriasis is one of the most common skin diseases, affecting over 125 million people worldwide. It is a chronic skin disease represented by red squamous plaques that usually appear on the elbows, knees, sacroiliac region, nails, and scalp. Its histological features are characterized by epidermal hyperplasia, increased angiogenesis, and immune cell infiltration (Zhang Y. et al., 2020). There is also an extensive list of comorbidities associated with this disease (including Crohn’s disease, psoriatic arthritis, atherogenic dyslipidemia, hypertension, diabetes, as well as increased carotid intima-media thickness) (Martinez-Lopez et al., 2018; Sanabria-de la Torre et al., 2020). It is fundamentally associated with immunological and genetic factors. Treatments are varied and tend to be aimed at reducing skin lesions; this is a current area of research. The therapeutic effects of MSCs on psoriasis have been reported in experimental studies and clinical cases (Chen et al., 2016; De Jesus et al., 2016; Sah et al., 2016). In two clinical cases of psoriasis vulgaris treated with MSCs, both of them remained relapse-free for 4 or 5 years.

As mentioned above, MSC exosomes have therapeutic effects on various relapsing inflammatory disorders such as AD and cGVHD. Accumulative evidence has indicated that MSC exosomes exhibit potent immunomodulatory effects by regulating the activation of immune cells and inhibiting the expression of various inflammatory cytokines (Kordelas et al., 2014; Cho et al., 2018; Lai et al., 2018).

In fact, Zhang Y. et al. (2020) demonstrated that hUC-MSC-derived exosomes prevented the progression and reduced the severity of psoriasis by regulating immune cells, through inhibiting *in vitro* the maturation and activation of **DCs** and Th17 cells, along with HaCaTs. In addition, treatment with these exosomes effectively blocked the induction of inflammatory cytokines and reduced both histopathological symptoms and immune responses in a mouse model *in vivo* (Table 1 and Supplementary Table 3).

## Exosomes and Melanoma

The tumor necrosis factor-related apoptosis-inducing ligand (TRAIL) is a member of the TNF family with a selective effect on cancer cells. As a promising agent for cancer therapy, TRAIL can induce cell apoptosis by interaction with its receptors, death receptor 4 and 5 (DR4 and DR5) on tumor cells. Human melanoma cells express TRAIL DR5 (Kurbanov et al., 2005). Murine melanoma cells (B16) express a moderate level of TRAIL-R on their cell surface (Dufour et al., 2017). Mueller et al. demonstrated that TRAIL-modified MSCs (MSC-TRAIL) could effectively induce apoptosis in sensitive advanced colorectal carcinoma cells *in vitro* and also inhibit tumor growth in animal models (Mueller et al., 2011). Yuan et al. (2017) showed that exosomes derived from MSC-TRAIL (TRAIL exosomes) were capable of inducing effective apoptosis in a wide range of cancer cell lines including lung adenocarcinoma, primary normal human bronchial epithelial cells, renal cancer cells, breast adenocarcinoma and neuroblastoma cell lines *in vitro*. Compared to recombinant soluble TRAIL, TRAIL exosomes exert a greater killing efficiency of cancer cells. Due to the fluidic nature of the bilayer membrane, the lipid membrane of TRAIL exosomes allows higher TRAIL oligomerization and subsequently higher clustering of its receptors (Yuan et al., 2017). Also, the low pH condition of tumor microenvironment potentially influenced exosome trafficking and uptake by cancer cells (Parolini et al., 2009).

In a recent study, Shamili et al. (2018) investigated the anti-tumor effect of TRAIL exosomes, drawing on the combined use of tumor-specific cytotoxicity of TRAIL and intrinsic properties of BM-MSC-derived exosomes on tumor cells. Specifically, the anti-tumor activity of MSC exosomes and TRAIL exosomes was analyzed *in vitro* and in three *in vivo* models (Table 1 and Supplementary Table 3).

*In vitro* analysis (Table 1) consisted of a co-culture of the exosomes with murine melanoma cells (B16F0 cells). There was a significant difference in cell viability between cells treated with TRAIL exosomes and those treated with MSC exosomes. Specifically, TRAIL exosomes and MSC exosomes induced 11.93 and 4.69% apoptosis in B16F0 cells, respectively (Shamili et al., 2018). To evaluate the *in vivo* anti-tumor activity of these exosomes (Supplementary Table 3), three models of melanoma tumor-bearing mice were developed. In the co-delivery model, tumor cells were co-injected with exosomes and in the non-co-delivery model exosomes were injected after tumor appearance in a single dose or in multiple doses. The co-delivery model resulted in a delayed appearance of the tumor for 6 days and a reduction in size. The non-co-delivery administration with a single dose showed anti-tumor activity in early days, delayed tumor growth and an increased life expectancy of 4 days. Finally, non-co-delivery administration with multiples doses also resulted in a significant reduction of tumor size and an increased life expectancy of 8 days. Delayed tumor growth was higher compared to the single doses. In summary, it was demonstrated that MSC exosomes and TRAIL exosomes have potential capacity as targeted cancer treatment, with the best option being TRAIL exosomes. Specifically, the best administration option was delivery after tumor appearance of TRAIL exosomes in multidose. These exosomes could be exploited for drug delivery purposes to deliver therapeutic agents. In addition, this study highlights the possibility of combining TRAIL exosomes and chemotherapeutics as a promising anti-tumor strategy (Shamili et al., 2018).

de Araujo Farias et al. (2018) investigated the role of exosomes derived from irradiated MSCs (irMSC exosomes) A375 melanoma cell line *in vitro* and in tumor growth and metastasis retardation after treatment with MSC + radiotherapy *in vivo* (Table 1 and Supplementary Table 3). In this study, the authors report the possible implication of exosomes cargo, specifically annexins (ANXA1 and ANAX2), integrins (ITGB1 and ITGA3), fibronectin 1 (FN1), catenin β1 (CTNNB1) and apolipoprotein H (APOH), in their suppressive action on A375 cells survival. Regarding *in vivo* outcomes, the ability of MSCs to accumulate at tumor sites makes them extremely attractive for targeted cancer therapy; in addition, the tumor tropism of MSCs has been reported to increase with radiation therapy (Kim et al., 2010). MSCs combined with radiation therapy enhances the effects of radiation on the metastatic spread of melanoma cells and exosome-derived factors could be involved in these effects (de Araujo Farias et al., 2018). MSC exosomes appear to bind to specific membrane microdomains in tumor cells, amplifying the action of radiation therapy, stimulating tumor cell death, thus increasing the sensitivity of cells to radiation and promoting systemic effects (Kim et al., 2010). Radiation therapy itself may not be systemic, although it could contribute to a systemic effect when used in combination with MSCs due to the ability of irMSC exosomes to increase the control of tumor growth and metastasis (de Araujo Farias et al., 2018).

## Discussion

Exosomes derived from MSCs are characterized by having immunomodulatory and regenerative properties and because of this they have gained much attention as a new potential cell-free approach in this field by overcoming the inherent limitations of the use of MSCs in the treatment of inflammatory skin diseases. Although the role of MSC exosomes in wound healing has been discussed (Roşca et al., 2018; Hu et al., 2019; Casado-Díaz et al., 2020), exosomes have been reported to play crucial roles in other skin conditions. In the present review, the function of MSC exosomes not only in wound healing and skin regeneration but also in oxidative stress and rejuvenation, hair growth, melanoma and autoimmune disorders such as GVHD, psoriasis and AD has been analyzed.

In this context, different signaling routes and molecules where exosomes are implicated have been proposed in the available bibliography, depending on the specific study. Regarding wound healing and skin regeneration, the most-reported signaling pathways include Wnt/β-catenin, PI3K/AKT, ERK, and TGF-β/Smad. Wnt/β-catenin and AKT cascades seem to participate in hair growth as well. Different miRNA and other molecules may also be involved. The NRF2 defense system is related to the oxidative stress process. With respect to autoimmune disorders, MSC exosomes seem to reduce pro-inflammatory cytokines and to promote induction of regulatory T cells. Finally, in melanomas, the administration of exosomes is capable of reducing tumor growth and size and promoting life span of *in vivo* models.

In addition to the mechanism of action, the origin of the exosomes can also affect their role. It is important to analyze the differences between fetal and adult MSC-derived exosomes. Firstly, regarding the source of MSCs, several aspects have to been taken into account. Commonly used adults MSCs as source of exosomes include AT-MSCs and BM-MSCs. AT-MSCs are usually isolated from left over biological material generated during liposuction, lipoplasty, or lipectomy procedures while BM-MSCs are obtained from bone marrow aspirate, an invasive and painful procedure (Hass et al., 2011). The most used fetal MSC source of exosomes is UC-MSCs. Other fetal MSCs include ESC-MSCs and FD-MSCs. Advantages derived from the use of these MSCs over adult MSCs comprise their ready availability and their obtaining from no invasive procedures. Furthermore, fetal MSCs have shown improved proliferative capacity, life span and differentiation potential (Hass et al., 2011). Considering the role of exosomes derived from both fetal and adult MSCs, several points need to be highlighted. Regarding fetal MSC-derived exosomes, UC-MSC-derived exosomes are implicated in wound healing, oxidative stress defense process, rejuvenation and psoriasis. No other source, fetal or adult, of exosomes has been used for testing its role in the oxidative stress defense process, rejuvenation and psoriasis. Regarding wound healing, FD-MSCs and iMSC (from Wharton’s Jelly derived IPSCs) have also been used. ESC-MSC-derived exosomes have showed beneficial effects in GVHD. Concerning adult MSC, AT-MSC-derived exosomes are implicated in wound healing and AD. No other source of MSC has been used for testing exosome’s role in AD. Among adult MSCs, just BM-MSC-derived exosomes have been shown improvements on acute, chronic and refractory GVHD, melanoma and hair growth. Men-MSC- and G-MSC-derived exosomes have improved wound healing outcomes. Comparative studies are lacking about specific differences between exosomes from fetal MSC and from adult MSC.

Regarding the possible clinical translation of exosomes, it is essential to determine the optimal source of MSCs. Cell senescence due to culture passages and time can alter the exosome cargos and therefore their properties. In this sense, iMSC is a strategy for exosome production with several advantages. Thus, established iPSCs can expand indefinitely and easily become iMSCs within 20 days. Moreover, the *in vitro* growth and differentiation potential of MSCs is affected by the culture period, the donor’s age and the donor’s health status, factors that are also resolved with the use of iMSC. Furthermore, iMSCs avoid ethical problems and immune rejection (Kim et al., 2018). In this sense, MSCs from oral cavities are more easily harvestable than other anatomic sites and have shown a great plasticity toward the main lineages, specifically toward bone tissues. Human periapical inflamed cyst in particular represents a promising source of MSCs because of the reduced ethical and biological issues as it is usually considered biological waste (Spagnuolo et al., 2018; Tatullo et al., 2019). Moreover, it is important to determine the optimal culture conditions. As mentioned throughout this review, it is feasible to condition MSC media in order to enhance determined exosome features depending on the study. In addition, the methodology for isolating and purifying exosomes must be highly controlled and optimized.

However, due to the lack of clinical trials, the heterogeneity of MSC products prepared by different laboratories, diverse strategies of MSC isolation and absence of standardization across groups, it is difficult to achieve the MSC exosome bench to bedside translation as any new therapeutic platform requires the establishment of GMP conditions and standards for production. In case of exosomes, there are some limitations to overcome. These limitations include large-scale production, downstream purification methods, and quality control systems (Jafari et al., 2020). Regarding limitations on exosome’s production, studies about scaling up cell culture have focused on technologies to maximize surface area (Colao et al., 2018). Hollow-fiber bioreactors, which are high-density systems for culturing cells, allow continuously production of exosomes for more than 10 weeks, without splitting or subculturing the cells, increasing the exosome production from 10 to 100 fold (Whitford et al., 2015; Colao et al., 2018; Jafari et al., 2020). Other strategies include genetic manipulation of MSCs for producing an immortalized cell line, which continuously produced exosomes or the design of PEGylated wells for the aggregation of cells and the formation of MSC spheroids to enhance cell-to-cell communication (Jafari et al., 2020). Once scaling up cell culture of MSC for large-scale production of exosomes is achieve, it is necessary to define them physically, biochemically and functionally by quantifiable features and using reproducible and standardized assays. Members of the Society for Clinical Research and Translation of Extracellular Vesicles Singapore (SOCRATES), the International Society for Extracellular Vesicles (ISEV), the International Society for Cell and Gene Therapy (ISCT) and the International Society of Blood Transfusion (ISBT) have proposed specific harmonization criteria for MSC-small EVs (sEVs) to facilitate data sharing and comparison (Witwer et al., 2019). Precisely, MSC-sEVs should be defined by quantifiable metrics to identify the cellular origin of the sEVs in a preparation, presence of lipid-membrane vesicles and the degree of physical and biochemical integrity of the vesicles. Therefore, specifications related to purity, identity, quantity, potency, and sterility need to be defined in accordance with the regulations for pharmaceutical manufacturing (Gimona et al., 2017). Quality can be assessed by producing them through validated procedures. Sterility testing is achieved by stablished protocols for detection of microbiological and endotoxin contaminations. Potency issues relies on more molecular biology and physiology information. In fact, qualified potency assays in disease-relevant *in vitro* and *in vivo* models are necessary to elucidate their mechanism of action (Gimona et al., 2017; Jafari et al., 2020). Limitations on downstream purification and characterization methods make it difficult to determine purity and identity of exosomes. In these aspects, heterogeneity is a critical issue. Research efforts are focus on strategies to improve the isolation of exosomes, although there is no consensus on the most suitable method for exosomes enrichment and purification (Gimona et al., 2017). Some recent technologies include serial filtration, tangential flow filtration (TFF) or polymer precipitation (Gimona et al., 2017; Colao et al., 2018; Jafari et al., 2020). Standardized GMP-compliant TFF systems are available on the market, offering the possibility of validated process control. Another promising strategy comprises sequential filtration followed by affinity-based chromatography that specific surface proteins (Jafari et al., 2020). Considering identity of exosomes, the ISEV has established a set of criteria for their proteomic identification with a minimal list of requirements: exosomes should (1) have transmembrane proteins to provide evidence of a membrane; (2) have cytosolic proteins to provide evidence of membrane- or receptor-binding; (3) be free of protein impurities from intracellular compartments not associated with the plasma membranes or endosomes; and (4) be free of co-isolating extracellular proteins (Colao et al., 2018). Characterization approaches of exosomes include nanoparticle tracking analysis (NTA) and dynamic light scattering (DLS) for size distribution and zeta potential determination, ELISA assay, Western blotting, and flow cytometry for exosome protein analysis and quantitative reverse transcription-PCR (qRT-PCR), mass spectrometry, and miRNA array for exosome content determination (Jafari et al., 2020). However, since exosomes are still difficult to purify, minimal acceptance and release criteria include these statements: (1) MSCs display an ISCT-compliant surface marker profile at the time of secretome harvest; (2) EVs within the secretome fraction must comply with the minimal criteria of ISEV, at least for a number of membrane markers; (3) The size range should be in the range of exosomes (50–150 nm) and (4) Sterility and endotoxin levels must comply with regulatory requirements (Gimona et al., 2017).

Another factor to consider is the *in vivo* administration of exosomes. As has been shown, subcutaneous and intravenous are the most frequently used administrations although it is possible to deliver them through different kinds of hydrogels. In this context, scaffolds have recently gained much attention. In fact, the geometrical and mechanical properties of scaffolds are able to influence the secretome, interactome and cell behavior (Ballini et al., 2017). Computer-aided design (CAD) has emerged as a promising strategy for biomimetic scaffold manufacturing as it allows the development of standardized design to test different types of scaffolds by reducing bias during measurements (Marrelli et al., 2016; Ballini et al., 2017). Thus, it is important to optimize the application of EVs for skin wound healing. Therefore, it would be convenient to optimize and analyze the advantages and disadvantages of the different application methodologies for each specific clinical objective.

In conclusion, the currently available data in this field shows how MSC exosomes can improve critical parameters of preclinical and clinical models of several skin conditions, including wound healing, oxidative stress, photoaging and rejuvenation, AD, psoriasis, GVHD and melanoma. Exosomes stimulate the migration and proliferation of fibroblast and keratinocytes, promote angiogenesis, collagen synthesis, re-epithelization and wound closure in wound healing, stimulate hair growth and have immunoregulatory effects on inflammatory skin diseases. Regarding oxidative stress, they are able to decrease ROS generation and DNA damage. Lastly, in melanoma, exosomes reduce tumor growth and promote life span. However, very few studies have reported their role on human skin disease models. Kwon et al. (2020) conducted a 12-week prospective, double-blind, randomized, split-face, comparative study to evaluate the clinical efficacy of application of hAT-MSC exosomes after fractional CO_2_ laser in the treatment of facial acne scars. The application of exosomes treatment yielded more favorable responses, a shorter recovery time, and fewer side-effects (Kwon et al., 2020). Regarding clinical trials, to date there are only two about MSC exosome treatment on humans (NCT04173650 and NCT04213248). The first trial will analyze the impact of MSC exosomes on dystrophic epidermolysis bullosa human wounds and the second their effect on dry eye symptoms in patients with cGVHD. However, both are still in the recruitment phase.

Therefore, further research is required for a better understanding of the molecular mechanisms underlying the action and therapeutic potency of MSC exosomes in the clinical context. In order to assess their clinical translation, methods for large-scale production should be optimized and standardized, including the source of MSCs, their isolation and culture conditions as well as the administration protocols for exosomes.

## Author Contributions

MQ-V had the conception, revised the bibliography, and wrote the manuscript. RS-DT and AF-G revised the bibliography and the different versions of the manuscript. MS-D, ÁS-S, and TM-V revised the different versions of the manuscript. SA-S had the conception, revised bibliography and the different versions of the manuscript. All the authors contributed to the article and approved the submitted version.

## Conflict of Interest

The authors declare that the research was conducted in the absence of any commercial or financial relationships that could be construed as a potential conflict of interest.

## Supplementary Material

Supplementary Table 1 *In vitro* studies of MSC exosomes in wound healing.

Supplementary Table 2 *In vivo* studies of MSC exosomes in wound healing.

Supplementary Table 3 *In vivo* studies of MSC exosomes in other skin conditions.

## Glossary

Glossary: AD, atopic dermatitis; AT-MSCs, adipose tissue mesenchymal stromal cells; aGVHD, acute graft versus host disease; AKT, protein kinase B; AIF, apoptosis-inducing factor; Ang-2, angiotensin-2; ANX, annexin; APOH, apolipoprotein H; ARG, arginine; ATV, atorvastatin; BM-MSCs, bone marrow mesenchymal stromal cells; BMP, bone morphogenetic protein signaling; CAD, computer-aided design; CCL20, C-C motif chemokine ligand 20; CTNNB1, catenin β1; cGVHD, chronic graft versus host disease; CXCL12, C-X-C motif chemokine 12; DAMPs, damage-associated molecular patterns; DCs, dendritic cells; DP, dermal papilla; DP-MSCs, dermal papilla mesenchymal stromal cells; DR, death receptor; ECM, extracellular matrix; ERK, extracellular signal-regulated kinase; EVs, extracellular vesicles; eNOS, endothelial nitric oxide synthase; ESCRT, endosomal sorting complex required for transport; Exos, exosomes; FD-MSCs, fetal dermal mesenchymal stromal cells; FGF, fibroblast growth factor; FN1, fibronectin 1; GMP, good manufacturing practice; G-MSCs, gingival mesenchymal stromal cells; GVHD, graft versus host disease; HaCaTs, human cultured keratinocytes; hAD-MSCs, human adipose tissue mesenchymal stromal cells; hAECs, human amniotic epithelial cells; hBM-MSCs, human bone marrow mesenchymal stromal cells; HDFs, human dermal fibroblasts; hESC-MSCs, human embryonic stem cell–derived MSCs; HGF, hepatocyte growth factor; HO-1, heme oxygenase 1; hsp, head shock protein; hUC-MSCs, human umbilical cord mesenchymal stromal cells; HUVECs, human umbilical vein endothelial cells; IFN-γ, interferon gamma; IGF, insulin-like growth factor; IL, interleukin; iMSCs, induced mesenchymal stromal cells; iNOS, inducible nitric oxide synthase; irMSCs, irradiated mesenchymal stromal cells; ISBT, International Society of Blood Transfusion; ISCT, International Society for Cell and Gene Therapy; ISEV, International Society of Extracellular Vesicles; ITG, integrin; Keap1, Kelch-like ECH-associated protein 1; KGF, keratinocyte growth factor; LATS, large tumor suppressor; LEF1, lymphoid enhancer-binding factor 1; MALAT1, metastasis associated lung adenocarcinoma transcript 1; MAPK, mitogen-activated protein kinase; Men-MSCs, menstrual blood mesenchymal stromal cells; MMPs, matrix metalloproteinases; MSCs, mesenchymal stromal cells; MSC-TRAIL, tumor necrosis factor-related apoptosis-inducing ligand modified mesenchymal stromal cells; mTOR, mammalian target of rapamycin; MVBs, multivesicular bodies; MWCO, molecular weight cutoff; NLRP3, NOD-like receptor protein 3 inflammasome; NOD-R, nucleotide-binding oligomerization domain-like receptors; NQO1, NAD(P)H quinone dehydrogenase 1; NRF2, nuclear factor E2-related factor 2; PAMPs, pathogen-associated molecular patterns; PBS, phosphate-buffered saline; PARP-1, poly (ADP ribose) polymerase 1; PCNA, proliferating cell nuclear antigen; PDGF-BB, platelet-Derived Growth Factor BB; PDGF-α, platelet-derived growth factor α; PF-127, pluronic F-127; PI3K/AKT, phosphatidylinositol 3-kinase/protein kinase B pathway; PTEN, phosphatase and tensin homolog; ROS, reactive oxygen species; α-SMA, α-smooth muscle actin; SHSs, *Haliclona* sp. spicules; STAT3, signal transducer and activator of transcription 3; SOCRATES, Society for Clinical Research and Translation of Extracellular Vesicles Singapore; Th, helper T cells; TGF-β1, transforming growth factor β1; TGF-β3, transforming growth factor β3; TIMPs, tissue inhibitor of matrix metalloproteinases; TLRs, toll-like receptors; TNF-α, tumor necrosis factor α; TRAIL, tumor necrosis factor-related apoptosis-inducing ligand; Tregs, regulatory T cells; TSG-6, tumor necrosis factor-inducible gene 6 protein; TSLP, thymic stromal lymphopoeitin; UC-MSCs, umbilical cord mesenchymal stromal cells; UV, ultraviolet; VEGF-A, vascular endothelial growth factor A; YAP, yes-associated protein.
