# Supplementary material for: The Role of Exosomes Derived From Mesenchymal Stromal Cells in Dermatology
Source: Front Cell Dev Biol. 2021 Apr 7;9:647012. doi: 10.3389/fcell.2021.647012 (PMC8058372; doi:10.3389/fcell.2021.647012)
Supplement: Supplementary Table 1 — In vitro studies of MSC exosomes in wound healing. [file Table_1.docx]

**Supplementary Table 1. *In vitro* studies of MSC exosomes in wound healing**

| Source of exosomes | Isolation Protocol | *In vitro* model | Skin condition | Signaling pathway and proteins involved | Outcomes | Reference |
| --- | --- | --- | --- | --- | --- | --- |
| hAT-MSCs | Centrifugation of the culture medium at 4°C, 300g for 10 min. The debris was removed by centrifuging at 4°C, 2000g for 10 min. Then the medium was ultracentrifuged at 4°C, 10,000g for 70 min twice | HaCaTs | Wound healing | PI3K/AKT  ↑miR-21 and p-AKT  ↓TGβ-1  ↑MMP-2 and 9  ↓TIMP-1 and 2 | ↑Migration and proliferation | (Yang et al., 2020a) |
| hAT-MSCs | Centrifugation of culture medium at 300g for 10 min. Filtration (0.22 μm filter) and ultracentrifugation of the supernatant at 120,000g for 10 h | BJ cells | Wound healing | ↑miR-135a  ↓LATS2  ↓N-cadherin and E-cadherin | ↑Migration and proliferation | (Gao et al., 2020) |
| hAT-MSCs | Using exosome isolation reagent (RiboBio, Guangzhou, China), exosomes were extracted from supernatants without cell-debris in accordance with the manufacturer’s guidelines | HaCaTs and HDFs exposed to H_2_O_2_ | Wound healing | AT-MSC-Exos containing MALAT1  Targets miR-124 and activates Wnt/β-catenin pathway. | ↑Migration and proliferation  ↓Apoptosis | (He et al., 2020) |
| hAT-MSCs  hBM-MSCs  hUC-MSCs | Serial centrifugation of the culture medium at 300g for 10 min, at 2,000g for 10 min and at 10,000g for 30 min at 4ºC. Exosomes were collected by centrifuge at 100,000 × g for 70 min at 4ºC | HaCaTs and HDFs | Wound healing | ↑Growth factors (VEGF-A, FGF-2, HFG, PDGF-BB, TGF- β) | ↑Migration and proliferation | (Hoang et al., 2020) |
| ATV-pretreated hBM-MSCs | Centrifugation of the culture medium at 300g for 5 min and 2000g for 20 min. Filtration (0.22 μm filter) and ultracentrifugation of the supernatant at 120,000g for 1.5 h twice | HUVECs | Wound healing | ↑VEGF, PDGF, EGF, bFGF and ANG1  ↑ miR-221-3p  Activation of AKT/eNOS route | ↑Migration, proliferation and angiogenesis | (Yu et al., 2020) |
| hBM-MSCs | The culture medium was filtered using a 0.1 μm filter. The supernatant was concentrated with a 100-kDa (molecular weight cutoff) MWCO membrane at 1,000g for 30 min. The supernatant was loaded onto a 30% sucrose/D2O cushion and ultracentrifuged at 100,000g for 3 h. Exosome-enriched fraction was washed and centrifuged at 1500g 30 min with 100-KDa MWCO 3 times. Purified exosomes were passed through a 0.22 μm filter | HaCaTs and HDFs | Wound healing | ↑PCNA positive cells | ↑Migration and proliferation | (Jiang et al., 2020b) |
| BM-MSCs pretreated with serum of neonatal mice | The culture medium was centrifuged at 2,000g for 10 minutes. Next, the supernatant was centrifuged at 10,000g for 30 min. The final supernatant was then ultracentrifuged at 100,000g for 70 minutes twice | Endothelial cells | Wound healing | AKT/eNOS  ↑p-AKT and p-eNOS | ↑Migration, proliferation and angiogenesis | (Qiu et al., 2020) |
| hUC-MSCs | Centrifugation of the culture medium at 300g at 4°C for 10 min. The supernatant was collected, centrifuged at 16,500g at 4°C for 20 min and passed through a 0.22 μm filter. The filtrate was centrifuged at 120,000g at 4°C for 90 min | HaCaTs | Wound healing | PARP1/AIF  ↑PARP-1 and PAR  Inhibition of nuclear translocation of AIF | ↑Migration and proliferation  ↓Apoptosis induced by H_2_O_2_ | (Zhao et al., 2020) |
| hUC-MSCs | The culture medium was centrifuged at 300g for 10 min. Then the supernatant was centrifuged at 2,000g for 30 min. The supernatant was centrifuged at 10,000g for 1 h and filtered with a 0.22 µm filter and the precipitates obtained were exosomes | HDFs | Scarless wound healing | TGF- β/Smad  ↑Col III  ↓Col I, α-SMA and Smad2/3 | Inhibition of dermal fibroblast-myofibroblast transition | (Hu et al., 2020) |
| hUC-MSCs | Ultrafiltration membrane with a 100-kDa MWCO to condense collected culture medium by centrifugation at 1,500g for 30 min. Filtration (0.22 µm filter) of the supernatant. Incubation with exosome isolation reagent at 4°C overnight and centrifugation at 1,500g for 15 min at 4°C | HUVECs | Wound healing | ↑Ang-2 | ↑Angiogenesis | (Liu et al., 2020) |
| hUC-MSCs | The culture medium was collected and ExoQuick-TC exosome extraction reagent was added to the supernatant at a ratio of 1:5. After overnight incubation at 4°C, the supernatant was discarded, and the mixture was centrifuged at 1500g for 5 min to remove all liquid | HUVECs | Wound healing | ↑VEGF and TGF-β1 | ↑Migration, proliferation and angiogenesis | (Yang et al., 2020b) |
| hUC-MSCs | Centrifugation of the culture medium at 1500 rpm for 15 min and filtration using a 0.22 μm syringe filter. The supernatant was passed through a 100-kDa molecular weight Amicon® Ultra-15 Centrifugal Filter Device and concentrated. Exosomes were isolated using an exoEasy Maxi kit according to the manufacturer’s instructions | HUVECs | Wound healing | ↑Cyclin D1, A2, VEGF-A, CXCL12 | ↑Migration, proliferation and angiogenesis | (Li et al., 2020b) |
| AT-MSCs | Differential centrifugation (not specified) | HUVECs | Wound healing | Not characterized | ↑Proliferation, migration and tube formation ability | (Wang et al., 2019a) |
| hAT-MSCs | Exosome isolation reagent (RiboBio, Guangzhou, China) in accordance with the manufacturer’s guidelines | H_2_O_2_ -pretreated HaCaTs | Wound healing | Wnt/β-catenin | ↑Migration and proliferation  ↓Apoptosis | (Ma et al., 2019) |
| DFO-preconditioned hBM-MSCs | Centrifugation of the culture medium for 10 min at 500g. Centrifugation of the supernatant for 20 min at 12,000g and filtration using a 0.22 µm filter. Ultracentrifugation for 70 min at 110,000g twice | HUVECs | Wound healing | PI3K/AKT  ↑miR-126  ↓PTEN | ↑Angiogenesis | (Ding et al., 2019) |
| hFD-MSCs | Centrifugation of the culture medium at 3,000g for 15 minutes. Filtration (0.22 μm filter) of the supernatant and transference to an Amicon® Ultra-15 10K Centrifugal Filter Unit to concentrate to 1/5 volume. Appropriate volume of ExoQuick-TC was added to the supernatant. Storage at 4°C overnight and centrifugation at 1500g for 30 minutes | HDFs | Wound healing | ↑Collagen, elastin and fibronectin  Notch/Jagged 1 | ↑Proliferation, migration and ECM secretion | (Wang et al., 2019c) |
| hAT-MSCs pretreated with H_2_O_2_ | The culture medium was centrifuged at 500g for 10 min. Then, the supernatant was centrifuged at 12,000g for 20 min followed by filtration through a 0.22 µm filter. The exosomes were then pelleted by ultracentrifugation at 110,000g for 70 min. The resulting pellet was further purified by resuspension in PBS and ultracentrifugation at 110,000g for 70 min | HUVECs | Skin regeneration | Not characterized | ↑Migration and proliferation | (Bai et al., 2018) |
| hAT-MSCs | Not specified | HDFs | Wound healing | ↑CD34, Collagen I and KGF | ↑Migration proliferation | (Choi et al., 2018) |
| hAT-MSCs | The culture medium was centrifuged at 300g for 10 min, 2000g for 10 min and 10,000g for 30 min. Exosomes were collected from the supernatants by centrifugation at 100,000g for 70 min (4°C) twice | HDFs | Wound healing | PI3K/AKT  ↑p-Akt/Akt  ↑Col I and III, MMP1, bFGF and TGF-β1 | ↑Migration and proliferation | (Zhang et al., 2018b) |
| iMSCs (from iPSCs originating from Wharton’s Jelly hMSCs) | The culture medium was centrifuged for 5 min at 900g, then the supernatant was centrifuged for an additional 1 h at 10,000g. The supernatants were filtered through 0.2 µm pore filters. Exosomes were isolated by ultracentrifugation at 100,000g for 2 h and the pellet was subsequently washed with PBS and ultracentrifugated | HaCaTs and HDFs | Skin regeneration | ↑p-ERK1/2 | ↑Migration proliferation  ↑Collagen secretion  ↑Fibronectin secretion by HaCaTs | (Kim et al., 2018) |
| hAECs | Centrifugation of the culture medium at 300g for 5 min. Filtration (0.22 μm filter) and ultracentrifugation of the supernatant at 100,000g for 12 h | HDFs | Wound healing | ↑MMP-1 | ↑Migration and proliferation  ↓Collagen I and III | (Zhao et al., 2017) |
| hAT-MSCs | Centrifugation of the culture medium at 3,000g for 15 min and filtration through a 0.22 µm filter. Supernatants were concentrated using 100 KDa molecular weight Amicon Ultra-15 Centrifugal Filter Devicest and then incubated with ExoQuick-TC exosome precipitation® solution overnight | HDFs | Wound healing | ↑N-cadherin, cyclin-1, PCNA, Col I and III | ↑Migration, proliferation and collagen synthesis | (Hu et al., 2016) |
| hUC-MSCs | The culture medium was centrifuged at 1,000g for 20 mins, at 2,000g for 20 min and at 10,000g for 20 min. The supernatant was then concentrated with 100-kDa MWCO membrane at 1,000g for 30 minutes. The concentrated supernatant was loaded onto a 30% sucrose/D2O cushion and then ultracentrifuged at 100,000g for 1 h. The exosome-enriched fraction was diluted with PBS and then centrifuged 3 times at 1,000g for 30 min using 100 KDa MWCO. The purified exosomes were filtrated on a 0.22 μm pore filter | HaCaTs | Wound healing | Hippo-LATS signal  Inhibition of YAP activity and Wnt/β-catenin pathway at high cell densities  14-3-3f stimulates p-YAP by promoting p-LATS-YAP interactions | Restricted cell proliferation under relatively high cell density conditions | (Zhang et al., 2016) |
| hUC-MSCs | Centrifugation of the culture medium at 300g for 10 min at 4°C. The supernatant was centrifuged at 16,500g for 20 minutes at 4°C and then filtered through a 0.22 µm filter. The filtrate was ultracentrifuged twice at 120,000g for 70 minutes at 4°C to pellet the exosomes | TGF-βI Induced Myofibroblast | Scarless wound healing | miR-21, 23a, 125b, 145  Inhibition of TGF-β1/Smad2 cascade | ↓Myofibroblast differentiation | (Fang, S. et al. 2016) |
| hBM-MSCs | The culture medium was centrifuged at 2,000g at 4ºC for 20 min. The supernatant was centrifuged for 30 min at 10,000g and 4ºC. The supernatant was ultracentrifuged at 100,000g at 4ºC for 70 min. Pellets were resuspended in PBS and ultracentrifuged again at 100,000g at 4ºC for 70 min twice | HDFs from normal and chronic wounds  Endothelial cells | Wound healing | Activation of STAT3, AKT, ERK1/2  ↑Cyclin D2  ↑Growth factors | ↑Migration proliferation  ↑Angiogenesis | (Shabbir et al., 2015) |
| hUC-MSCs | The culture medium was centrifuged at 300g for 10 mins, at 2,000g for 10 min and at 10,000g for 30 min. The supernatant was then concentrated with 100-kDa MWCO membrane at 1,000g for 30 minutes. The concentrated supernatant was loaded onto a 30% sucrose/D2O cushion and then ultracentrifuged at 100,000g for 3 hours. The exosome-enriched fraction was collected and washed 3 times with PBS by centrifugation at 1,500g for 30 minutes with 100-KDa MWCO. Exosomes were passed through a 0.22 µm filter | Endothelial cells | Wound healing | Wnt/β-catenin  Delivered Wnt4 induces β-catenin activation  ↑PCNA, cyclin D3 and N-cadherin  ↓E-cadherin | ↑Migration proliferation and tube formation | (Zhang et al., 2015b) |
