# Supplementary material for: The Role of Exosomes Derived From Mesenchymal Stromal Cells in Dermatology
Source: Front Cell Dev Biol. 2021 Apr 7;9:647012. doi: 10.3389/fcell.2021.647012 (PMC8058372; doi:10.3389/fcell.2021.647012)
Supplement: Supplementary Table 2 — In vivo studies of MSC exosomes in wound healing. [file Table_2.docx]

**Supplementary Table 2. *In vivo* studies of MSC exosomes in wound healing**

| Source of exosomes | Isolation protocol | *In vivo* model | Administration | Skin condition | Signaling pathways and proteins involved | Outcomes | Reference |
| --- | --- | --- | --- | --- | --- | --- | --- |
| hAT-MSCs | Centrifugation of the culture medium at 4°C, 300g for 10 min. The debris was removed by centrifuging at 4°C, 2000g for 10 min. Then the medium was ultracentrifuged at 4°C, 10,0000g for 70 min twice | Full thickness skin wound mouse model | Injected (not specified) | Wound healing | ↑miR-21 | ↓Wound area (<50 % at d7 and <5 % at d14) | (Yang et al., 2020a) |
| hAT-MSCs | Centrifugation of the culture medium at 300g for 10 min. Filtration (0.22 μm filter) and ultracentrifugation of the supernatant at 120,000g for 10 h | Full-thickness skin  wound rat model | Subcutaneous | Wound healing | ↑miR-135a | ↓Healing area (<2 cm^2^ at d5, <1 cm^2^ at d5 and 15) | (Gao et al., 2020) |
| mmu_circ_0000250 modified-AT-MSCs | The culture medium was centrifuged at 300g for 10 min and again at 2,000g for 10 min. After centrifugation at 10,000g for 30 min, the supernatant was filtered (0.22 µm) and transferred to an Amicon® Ultra-15 Centrifugal Filter Unit (100 kDa) and centrifuged at 4,000g. The ultrafiltration unit was washed twice with PBS and the supernatant filtered again at 100,000g | Full-thickness skin excision diabetic rat model | Subcutaneous | Diabetic wound healing | ↑miR-128-3p  ↑SIRT1 | ↑Microvascular development (>3 fold blood vessel density increment)  ↓Apoptosis of skin tissue (<0.03% of relative apoptosis rate) | (Shi et al., 2020) |
| TSG-6 modified hBM-MSCs | Cell culture was centrifuged at 2,000g for 30 min and total exosome isolation was added to the collected culture medium and incubated at 4ºC overnight. Exosomes were extracted via centrifugation at 10,000g for 1 h at 4º C | Full thickness skin wound mouse model | Subcutaneous | Scarless wound healing | TGF- β/Smad  ↓TGF- β1  ↓p-Smad2 and 3 | ↓Inflammation  ↓Collagen deposition during scar formation | (Jiang et al., 2020a) |
| hBM-MSCs | The culture medium was first filtered using a 0.1 μm filter. The supernatant was concentrated with a 100-kDa MWCO membrane at 1,000g for 30 min. The supernatant was loaded onto a 30% sucrose/D2O cushion and ultracentrifuged at 100,000g for 3 h. Exosome-enriched fraction was centrifuged at 1,500g for 30 min with 100-KDa MWCO 3 times. Purified exosomes were passed through a 0.22 μm filter | Full thickness skin wound rat model | Subcutaneous | Scarless wound healing | TGF- β/Smad  ↓TGF-β1 and Smad2, 3, 4  ↑TGF-β3 and Smad 7  ↑VEGF-A and α-SMA | ↓Wound area from 60% to 10% at d8, 5% at d12 and 0% at d16)  ↑Cutaneous appendages (hair follicles and sebaceous glands) | (Jiang et al., 2020b) |
| BM-MSCs pretreated with serum of neonatal mice | Cell culture supernatant was centrifuged at 2,000g for 10 minutes. The supernatant was centrifuged at 10,000g for 30 min. The final supernatant was then ultracentrifuged at 100,000g for 70 minutes twice | Full thickness skin wound mouse model | Intradermic | Wound healing | ↑ KRT14 and CD31  ↓α-SMA | ↓Wound size (approx. 25% at d7 and 10% at d14)  ↑Angiogenesis (approx. 20% of CD31^+^ vessels)  ↓Fibrosis | (Qiu et al., 2020) |
| ATV-pretreated hBM-MSCs | Centrifugation of the culture medium at 300g for 5 min and 2,000g for 20 min. Filtration (0.22 μm filter) and ultracentrifugation of the supernatant at 120,000g for 1.5 h twice | Full-thickness skin excision diabetic rat model | Subcutaneous | Diabetic wound healing | ↑CD31 and α-SMA | ↑Wound healing rate (>80% at d7 and >90% at d14)  ↓Wound length (>0.4 cm at d7 and >0.2 cm at d14)  Neovascularization (<20 vessels/mm^2^ at d7 and approx. 50 vessels/mm^2^ at d14) | (Yu et al., 2020) |
| hUC-MSCs | Centrifugation of culture medium at 300g at 4°C for 10 min. The supernatant was collected, centrifuged at 16,500g at 4°C for 20 min and passed through a 0.22 μm filter. The filtrate was centrifuged at 120,000g at 4°C for 90 min | Full thickness skin wound rat model | Subcutaneous | Wound healing | ↑CD31 and CK10  ↓ α-SMA | ↑Wound healing (41.12% at d7 and 98.41% at d14)  ↑Dermal angiogenesis (approx. 60 new vessels/mm^2^ at d7 and 80 at d14)  ↓Scar formation (>50 µg of newly generated epidermis thickness at d7 and approx. 125 µg at d14) | (Zhao et al., 2020) |
| hUC-MSCs | Ultrafiltration membrane with a 100-kDa MWCO to condense collected culture medium by centrifugation at 1,500g for 30 min. Filtration (0.22 µm filter) of the supernatant. Exosome isolation reagent was added, incubated at 4°C overnight and centrifugation at 1,500g for 15 min at 4°C | Deep second-degree skin burn rat model | Subcutaneous | Burn wound healing | ↑Ang-2 | ↑Wound closure (>20% at d5, >40% at d9 and 80% at d13) | (Liu et al., 2020) |
| hUC-MSCs | Culture medium was collected and ExoQuick-TC exosome extraction reagent was added to the supernatant at a ratio of 1:5. After incubation overnight at 4°C, the supernatant was discarded and the mixture was centrifuged at 1,500g for 5 min to remove all liquid | Full thickness diabetic skin wound rat model | PF-127 topical hydrogel | Diabetic wound healing | ↑VEGF and TGF- β1  ↑CD31 and ki67 | ↓Residual wound area to approx. 10% at d10 and 5% at d14  ↑Regeneration of tissue granulation | (Yang et al., 2020b) |
| hUC-MSCs | Centrifugation of the culture medium at 1,500 rpm for 15 min and filtration using a 0.22 μm syringe filter. The supernatant was passed through a 100-kDa molecular weight Amicon® Ultra-15 Centrifugal Filter Device and concentrated. Exosomes were isolated using an exoEasy Maxi kit according to the manufacturer’s instructions | Full thickness skin wound rat model | Intravenous (exos + NPs with magnetic guidance) | Wound healing | ↑CK19, PCNA and Collagen | ↑Wound closure rate (approx. 40% at w1, >80% at w3 and 100% at w5)  ↓Wound edge length to <1 mm  ↑Collagen deposition area to >40% (similar to the control)  ↑Angiogenesis (approx. 25 vessels per field and approx. 15 mature vessels per field) | (Li et al., 2020b) |
| AT-MSCs | Differential centrifugation (not specified) | Full-thickness diabetic wound mouse model | FHE-exo hydrogel injection | Diabetic wound healing | ↑ ki67 and α-SMA expression | ↑Wound closure rates (>60% at d7, >80% at d14 and 100% at d21)  ↑Angiogenesis and re-epithelization  Well-organized collagen fibers at day 7 | (Wang et al., 2019a) |
| DFO-preconditioned hBM-MSCs | Centrifugation of the culture medium for 10 min at 500g. Centrifugation of the supernatant for 20 min at 12,000g and filtration using a 0.22 µm filter. Ultracentrifugation for 70 min at 110,000g twice | Full-thickness skin excision diabetic rat model | Subcutaneous | Diabetic wound healing | ↑CD31 and α-SMA | ↑Wound closure (approx. 90% at d7 and 95% at d14)  ↓Scar width (< 2mm at d14)  Neovascularization on wound sites (>30 vessels, approx. 15 mm^2^ of mature blood vessels at d14)  ↑Wavy collagen fibers | (Ding et al., 2019) |
| hFD-MSCs | Centrifugation of the culture medium at 3,000g for 15 minutes. Filtration (0.22 μm filter) of the supernatant and transference to an Amicon® Ultra-15 10K Centrifugal Filter Unit to concentrate to 1/5 volume. Appropriate volume of ExoQuick-TC was added in the supernatant. Storage at 4°C overnight and centrifugation at 1500g for 30 minutes | Full thickness skin wound mouse model | Subcutaneous | Wound healing | ↑PCNA and CK19 | ↓Wound size (approx. 0.4% at d7 and 0.1% at d14)  ↑Cell proliferation, ECM deposition, and re-epithelialization | (Wang et al., 2019c) |
| hMen-MSCs | Culture medium was centrifuged at 300g for 10 min, 2,000g for 20 min and 10,000g for 30 min at 4°C. Exosomes were precipitated using 100,000g ultracentrifugation for 60 min at 4°C | Full thickness diabetic skin wound rat model | Intradermic | Diabetic wound healing | ↑VEFG-A  ↑Collagen III/I ratio  ↑ARG:iNOS ratio  ↑*Rela* transcription at d7 post wounding (NF-κβ signaling) | ↑Angiogenesis (approx. 55 vessels per mm^2^ at d7 and 45 at d14)  ↑Wound closure (approx. 30% at d8 and 90% at d14)  ↑Re‐epithelialization (approx. 30% at d7 and 100% at d14)  ↓Relative fold of scar to approx. 0.5 of width, <0.5 of length and <0.2 of area  ↑M1 to M2 macrophage transition | (Dalirfardouei et al., 2019) |
| hAT-MSCs pretreated with H_2_O_2_ | Culture medium was centrifuged at 500g for 10 min. Then the supernatant was centrifuged at 12,000g for 20 min followed by filtration through a 0.22 µm filter. The exosomes were then pelleted by ultracentrifugation at 110,000g for 70 min. The resulting pellet was further purified by resuspension in PBS and ultracentrifugation at 110,000g for 70 min | I/R injury of flap transplantation model | Subcutaneous | Skin regeneration | Not characterized | ↑Flap survival ratio (>70% at d5 compared to <10% in I/R controls)  ↑Capillary density (approx. 100 blood perfusion units at d5 compared to 50 in I/R controls) (>35 microvessels per field at d5 compared to >5 in I/R controls)  ↓Apoptosis in the skin flap (20% compared to 90% in I/R controls) | (Bai et al., 2018) |
| hAT-MSCs | Culture medium was harvested at 4°C, with centrifugation steps at 300g for 10 min, 2,000g for 10 min and 10,000g for 30 min. Exosomes were collected from the supernatants by centrifugation at 100,000g for 70 min (4°C) 3 times | Full thickness skin wound mouse model | Subcutaneous and intradermic | Wound healing | ↑CD31 | ↓Wound area (50% at d7 and approx. 20% at d14)  ↓Scar width (>1 mm)  ↑Angiogenesis (approx. 60 mature vessels per mm^2^) | (Zhang et al., 2018b) |
| hAECs | Centrifugation of the culture medium at 300g for 5 min. Filtration (0.22 μm filter) and ultracentrifugation of the supernatant at 100,000g for 12 h | Full-thickness excisional skin wound rat model | Subcutaneous | Scarless wound healing | Not characterized | ↓Wound size (60% at d7, <5 % at d14 and <1% at d21)  ↑Degree of tissue reorganization | (Zhao et al., 2017) |
| hAT-MSCs | Culture medium was centrifuged at 3000 RCF for 15 min. Supernatants were passed through a 100 kDa molecular weight Amicon. The filtrate was passed through a 0.22 µm filter. ExoQuick-TC was added to the concentrated filtrate and refrigerated overnight at 4°C. The mixture was centrifuged at 1500 RCF for 30 min. The exosome-enriched pellets were resuspended in PBS and then passed through a 0.22 µm filter | Murine incisional wound model | Intravenous | Scarless wound healing | ↑TGF- β3 ↓TGF- β1  ↑Col III ↓Col I  ↓α-SMA  Activation of ERK/MAPK route  ↑MMP3 ↓TIMP1 | ↓Scar formation (<2 mm of width and approx. 0.2 mm of depth)  ↓Fibroblast into myofibroblasts differentiation  ↑ECM remodeling | (Wang et al., 2017) |
| BM-MSCs | Not specified | Full thickness skin defect dog model | Subcutaneous | Wound healing | Not characterized | ↑Wound reduction size (40% at d7 and 72.5% at d14)  ↑Collagen synthesis  ↑Neovascularization | (El-Tookhy et al., 2017) |
| hG-MSCs | The culture medium was centrifuged and passed through a 0.22 µm filter. The supernatant was then concentrated with 30 kDa MWCO hollow fiber membrane at 5,000g, 4ºC for 30 min. A volume of 0.5ml of clarified supernatant was flowed through the qEV column and eluted with PBS. The fractions from the supernatant were collected and concentrated again using the MWCO membrane | Full-thickness skin excision diabetic rat model | Topical chitosan/silk hydrogel | Diabetic wound healing | Not characterized | ↑Wound closure rate (approx. 60% at d7 and 90% at d14)  ↑Neoepithelization length (>6 mm at d7 and >9 mm at d14)  ↑Percentage of collagen fibers (approx. 60% at d7 and 70% at d14)  ↑Microvessel density (>40 vessels per field at d7 and approx. 50 at d14)  ↑Nerve density (>1 mm^2^ of neurofilament per field at d14) | (Shi et al., 2017) |
| hAT-MSCs | Centrifugation of the culture medium at 3,000g for 15 min, and filtration through a 0.22 um filter. Supernatants were concentrated using 100 KDa molecular weight Amicon® Ultra-15 Centrifugal Filter Devicest and then incubated with ExoQuick-TC exosome precipitation® solution overnight | Full-thickness wound mouse model | Intravenous (IV) and subcutaneous (SC) | Scarless wound healing | ↑Collagen I and III in early stage  ↓Collagen deposition in late stage | ↑Wound closure (approx. 30% at d7, 75% d14 and 90% d21 in IV administration and approx. 25% at d7, 60% at d14 and 80% at d21 in SC administration) | (Hu et al., 2016) |
| hUC-MSCs | Centrifugation of culture medium at 300g for 10 minutes at 4°C. The supernatant was centrifuged at 16,500g for 20 minutes at 4°C and then filtered through a 0.22 µm filter. The filtrate was ultracentrifuged twice at 120,000g for 70 minutes at 4°C to pellet the exosomes | Full thickness skin wound rat model | Subcutaneous hydrogel | Scarless wound healing | ↓α-SMA expression  ↑miR-21, 23a, 125b, 145  ↓p-Smad2  Inhibition of TGF- β1/Smad2 cascade | ↓Fibrosis  ↓Scar formation | (Fang, S. et al. 2016) |
| hUC-MSCs | The culture medium was centrifuged at 1,000g for 20 mins, at 2,000g for 20 min, and at 10,000g for 20 min. The supernatant was then concentrated with 100-kDa MWCO membrane at 1,000g for 30 minutes. The concentrated supernatant was loaded onto a 30% sucrose/D2O cushion and then ultracentrifuged at 100,000g for 1 h. The exosome-enriched fraction was diluted then centrifuged 3 times at 1,000g for 30 min using 100 KDa MWCO. The purified exosomes were filtrated on a 0.22 μm pore filter | Deep second degree burn rat model | Subcutaneous | Burn wound healing | ↑PCNA after 1-2 weeks  ↓PCNA, β-catenin, α-SMA and collagen after 4 weeks  14-3-3f protein restricts excessive cell expansion and  collagen deposition | Restricted excessive skin cell expansion and collagen deposition at late stage | (Zhang et al., 2016) |
| hUC-MSCs | The culture medium was centrifuged at 1,000g for 20 mins, at 2,000g for 20 min and at 10,000g for 20 min. The supernatant was then concentrated with 100-kDa MWCO membrane at 1,000g for 30 minutes. The concentrated supernatant was loaded onto a 30% sucrose/D2O cushion and then ultracentrifuged at 100,000g for 1 h. The exosome-enriched fraction was diluted and then centrifuged 3 times at 1,000g for 30 min using 100 KDa MWCO. The purified exosomes were filtrated on a 0.22 μm pore filter | Deep second degree burn rat model | Subcutaneous | Burn wound healing | ↑CK19, PCNA, collagen I  Activation of Wnt/β-catenin and AKT signaling | ↑Cell proliferation and Re-epithelization | (Zhang et al., 2015a) |
| hUC-MSCs | Culture medium was centrifuged at 300g for 10 mins, at 2,000g for 10 min, and at 10,000g for 30 min. The supernatant was then concentrated with 100-kDa MWCO hollow fiber membrane at 1,000g for 30 minutes. The concentrated supernatant was loaded onto a 30% sucrose/D2O cushion and then ultracentrifuged at 100,000g for 3 hours. The exosome-enriched fraction was collected and washed by centrifugation at 1,500g for 30 minutes with 100-KDa MWCO. Exosomes were passed through a 0.22 µm filter | Deep second-degree skin burn rat model | Subcutaneous | Wound healing | ↑CD31 | ↑Angiogenesis (>2 fold increment in tube length at 80µg/mL and >3 fold at 160µg/mL) | (Zhang et al., 2015b) |
