# Supplementary material for: The Role of Exosomes Derived From Mesenchymal Stromal Cells in Dermatology
Source: Front Cell Dev Biol. 2021 Apr 7;9:647012. doi: 10.3389/fcell.2021.647012 (PMC8058372; doi:10.3389/fcell.2021.647012)
Supplement: Supplementary Table 3 — In vivo studies of MSC exosomes in other skin conditions. [file Table_3.docx]

**Supplementary Table 3. *In vivo* studies of MSC exosomes and other skin conditions**

| Source of exosomes | Isolation Protocol | *In vivo* model | Administration | Disease | Signaling pathway and proteins involved | Outcomes | Reference |
| --- | --- | --- | --- | --- | --- | --- | --- |
| hAT-MSCs | The culture medium was filtrated through a 0.22 µm polyethersulfone membrane filter and then concentrated by tangential-flow filtration with a 500 kDa MWCO filter membrane cartridge. Buffer exchange was performed by diafiltration with PBS | Murine model of atopic dermatitis | Subcutaneous | Atopic dermatitis | ↓IL-4, IL-5, IL-13, IL-17, TNF-α, INF-γ and TSLP  ↑epidermal ceramides | ↓Skin inflammation  ↑Significant improvement in epidermal barrier functions  ↓Epidermal hyperplasia and erythema (120 µm with 1 µg, 100 µm with 3 µg and 80 µm with 10 µg)  ↓TEWL in a dose-dependent manner (50 g/m^2^/h with 1 µg, 25 g/m^2^/h with 3 µg and 30 g/m^2^/h with 10 µg)  ↑Stratum corneum hydration (11 units with 1 µg, 19 units with 3 µg, 25 units with 10 µg) | (Shin et al., 2020) |
| hUC-MSCs | The culture medium was processed using a series of centrifugation steps (300g for 10 min, 2,000g for 10 min and 10,000g for 30 min). Exosomes were collected via ultracentrifugation at 100,000g for 70 min | UV- irradiated mouse model | Intracutaneously | Oxidative stress | ↓TNFα, IL-1β and IL-6  ↓CK14, Ki67, P53, P21  ↓Col I and III  ↓NRF2, Keap1, HO-1 and NQO1  (NRF2 defense system) | Attenuated histological injury and inflammatory responses  Prevention of cell proliferation and collagen deposition | (Wang et al., 2020) |
| hUC-MSCs | The culture medium was successively centrifuged at 400g for 10 min, 2000g for 30 min and 10,000g for 60 min. The supernatant was passed through a syringe filter (0.22 μm) and centrifuged at 100,000g for 120 min to pellet exosomes. The pellet was washed and centrifuged for another 120 min at the same high speed | UV-irradiated mouse model and guinea pig | Topical (exosomes + SHSs) | Rejuvenation | ↑Collagen I, elastin and fibronectin  ↓MMP-1 | ↑Skin penetration (dermis and epidermis) compared to exosomes alone  ↓Microwrinkles  ↑Expression of ECM constituents  ↓Skin irritation (guinea pigs) | (Zhang et al., 2020a) |
| hUC-MSCs | The culture medium was centrifuged at 300g for 10 minutes and 16,500g for 30 minutes, and then filtered through a 0.22 µm filter. The final supernatant was then ultracentrifuged at 100,000g for 70 minutes to pellet exosomes. The pellet was filtered through a 0.22 µm filter and centrifuged at the same speed | Murine model of psoriasis | Subcutaneous | Psoriasis | ↓STAT3/pSTAT3  ↓IL-17, IL-23 and CCL20 | Ameliorated symptoms and diminished the clinical and pathological scores of psoriasis  <50% psoriasis area and severity index  ↓epidermis thickness (80 to 30 µm) | (Zhang et al., 2020b) |
| hAT-MSCs | Centrifugation and filtration through a 0.22 μm filter of the culture medium. Exosomes were concentrated and purified by tangential flow filtration with MWCO of 300 or 500 kDa | Murine model of atopic dermatitis | Subcutaneous and intravenous | Atopic dermatitis | ↓IgE in serum  ↓mast cell infiltration  ↓eosinophils  ↓IL-4, IL-31, IL-23, TNF-α | ↓Significantly decreased atopic dermatitis symptoms in a dose-dependent manner (100% clinical improvement with 0.14 µg, 150% with 1.4 µg and 220% with 10 µg)  ↓Significantly decreased ear thickness (500 to 700 µm comparing mice treated with prednisolone and mice treated with MSC exosomes) | (Cho et al., 2018) |
| hBM-MSCs | The culture medium was centrifuged at 2,000g for 30 minutes. The supernatant was passed through a 0.2 μm filter, mixed with Total Exosome Isolation Reagent, incubated overnight and centrifuged at 10,000g for 1 h | Murine model of GVHD | Intravenous | aGVHD | ↓CD4+ and CD8+ T cells  ↓ratio of CD62L-CD44+ to CD62L+CD44- T cells  ↑CD4+CD25+FoxP3+ regulatory T cells | ↓Reduced the pathological damage in multiple GVHD-targeted organs  ↑Prolonged survival (Controls 0% survival at d20; treated 15% survival at d100)  ↓Clinical score (from 3.5 to 2.8 points (on a scale of 10))  ↓Severe skin damage (from 88.8% to 60%) | (Fujii et al., 2018) |
| hBM-MSCs | The culture medium was centrifuged at 200g for 10 min, 2,000g for 20 min, 10,000g for 30 min and 110,000g for 7 h at 4°C, followed by filtration using a 0.22 μm filter. The culture supernatant was collected and ultracentrifugation performed with the same sequential centrifugation procedure. The pellet was washed twice with PBS and then filtered through a 0.22 μm filter | Murine model of cGVHD | Intravenous | cGVHD | Inhibition of IL-17 expressing pathogenic T cells  Induction of IL-10 expressing regulatory cells | ↓Clinical and pathological scores of cGVHD  Ameliorated fibrosis in the skin, lung and liver (<30% fibrosis of the skin)  Immunomodulatory effects  ↑Survival (from 50 to 60 days after engraftment)  ↓Clinical score (from 6 to 3 points on d50 after engraftment (on a scale of 7.9))  ↓Skin score (from 3.8 to 2.5 points on d50 after engraftment (on a scale of 3.9)) | (Lai et al., 2018) |
| BM-MSCs | The culture medium was filtered through 0.22 µm pore size membrane and subjected to three continuous centrifugations at 300g (10 min), 1,200g (30 min) and 10,000g (45 min). Then the supernatant was concentrated using 100 kDa Amicon® filter and exosomes were isolated with Exoquick TC kit according to the protocol | Murine model of melanoma | Subcutaneous (codelivery model) and Intravenous (in a single dose (ISD) and in multidose (IMD)) (Non-codelivery models) | Melanoma | Not characterized | Delayed tumor appearance  ↓Tumor growth (13.29% with IMD and 6.69 % with ISD)  ↑Median survival time (29 days with IMD and 25 days with ISD)  ↑Life span (38.09% with IMD and 19% with ISD)  ↓Tumor size (150 mm^3^ of tumor size at d24 with subcutaneous injection, 1800 mm^3^ of tumor size at d24 with ISD and 1200 mm^3^ of tumor size at d23 with IMD)  Codelivery model delayed tumor appearance for 6 days | (Shamili et al., 2018) |
| hESC-MSCs | The culture medium was 0.22-µm filtered and concentrated 100 × for exosomes by tangential flow filtration (MWCO 100 kDa) | Murine model of GVHD | Intravenous | GVHD | ↑Treg | Alleviated and significantly improved GHVD symptoms and survival  ↑Prolonged survival (30% controls at d23, 70% with 1 µg exosomes at d23 and 75% with 10 µg exosomes at d23), (0% controls at d30, 38% with 1 µg exosomes at d30 and 46% with 10 µg exosomes at d30)  ↓Mean combined grade (loss of weight, posture, activity, appearance of coat, integrity of the skin and paleness) (8.0 controls (on a scale of 10), 3.5 with 1 µg exosomes, 5.0 with 10 µg exosomes at d27) | (Zhang et al., 2018a) |
| hBM-MSCs | The culture medium was centrifuged at 300g for 10 min, at 1,500g for 20 min and finally at 2,500g for 20 min. The supernatant was filtered through a 0.2 µm syringe filter and ultracentrifuged at 100,000g for 60 min. Pellets were washed and ultracentrifuged again | Rats | Subcutaneous | Hair growth | ↑Phosphorylation of AKT, ERK and Bcl-2  ↑VEGF, IGF-1  ↑Wnt 3a and 5a | Transform the hair follicles from the telogen to the anagen phase  DP cells proliferation increased 1.5 times and migration rate was tripled  ↑60% hair regrowth at d18 and ↑100% at d27. | (Rajendran et al., 2017) |
| hBM-MSCs | Polyethylenglycol (PEG)-based protocol was used to enrich for the EV fractions of the supernatants. To remove residual polyethylene glycol and soluble proteins, pellets were washed with 0.9% NaCl and reprecipitated at 100,000 g for 2 h | Preliminary clinical study (human model) | Intravenous | Refractory GVHD | ↓IL1β, TNF-α and INF- γ | Remarkable cutaneous and mucosal response  Improved GVHD clinical symptoms | (Kordelas et al., 2014) |
| hESC-MSCs | The culture medium was concentrated 50x by tangential flow filtration using a mem- brane with a 100 kDa MWCO and then fractionated by high-performance liquid chromatography. The first eluted peak that contained the exosomes was concentrated using a 100 kDa MWCO filter | Murine model of GVHD | Intrasplenic | GVHD | ↑MYD88-dependent signaling  ↑Induction M2-like phenotype in monocytes  ↑Treg | Delayed graft rejection  Immunomodulatory effects  ↓Mean rejection score from 3.5 points to 1.6 points at d11 (on a scale of 6) and from 5 points to 4.5 points at d15 | (Zhang et al., 2014) |
